# Distinct Endophytic Bacterial Communities Inhabiting Seagrass Seeds

**DOI:** 10.3389/fmicb.2021.703014

**Published:** 2021-09-21

**Authors:** Flavia Tarquinio, Océane Attlan, Mathew A. Vanderklift, Oliver Berry, Andrew Bissett

**Affiliations:** ^1^Oceans and Atmosphere, Indian Ocean Marine Research Centre, Commonwealth Scientific and Industrial Research Organisation (CSIRO), Crawley, WA, Australia; ^2^Environomics Future Science Platform, Indian Ocean Marine Research Centre, Commonwealth Scientific and Industrial Research Organisation (CSIRO), Crawley, WA, Australia; ^3^Sciences et Technologies, Université de la Réunion, Saint-Denis, France; ^4^Oceans and Atmosphere, Commonwealth Scientific and Industrial Research Organisation (CSIRO), Hobart, TAS, Australia

**Keywords:** *Halophila ovalis*, seagrass restoration, seed microbiome, core, bacteria, amplicon sequencing

## Abstract

Seagrasses are marine angiosperms that can live completely or partially submerged in water and perform a variety of significant ecosystem services. Like terrestrial angiosperms, seagrasses can reproduce sexually and, the pollinated female flower develop into fruits and seeds, which represent a critical stage in the life of plants. Seed microbiomes include endophytic microorganisms that in terrestrial plants can affect seed germination and seedling health through phytohormone production, enhanced nutrient availability and defence against pathogens. However, the characteristics and origins of the seagrass seed microbiomes is unknown. Here, we examined the endophytic bacterial community of six microenvironments (flowers, fruits, and seeds, together with leaves, roots, and rhizospheric sediment) of the seagrass *Halophila ovalis* collected from the Swan Estuary, in southwestern Australia. An amplicon sequencing approach (16S rRNA) was used to characterize the diversity and composition of *H. ovalis* bacterial microbiomes and identify core microbiome bacteria that were conserved across microenvironments. Distinct communities of bacteria were observed within specific seagrass microenvironments, including the reproductive tissues (flowers, fruits, and seeds). In particular, bacteria previously associated with plant growth promoting characteristics were mainly found within reproductive tissues. Seagrass seed-borne bacteria that exhibit growth promoting traits, the ability to fix nitrogen and anti-pathogenic potential activity, may play a pivotal role in seed survival, as is common for terrestrial plants. We present the endophytic community of the seagrass seeds as foundation for the identification of potential beneficial bacteria and their selection in order to improve seagrass restoration.

## Introduction

It is well established that vascular plants form complex interactions and mutualistic relationships with bacteria that play a critical role in supporting plant growth and fitness (Turner et al., 2013; Fitzpatrick et al., 2018). This work, conducted largely on terrestrial vascular plants, has demonstrated that such diverse functions as boosted growth and protection from pathogens, can be attributed to interactions between plants and their unique microbial communities (microbiomes; Compant et al., 2005; Gopalakrishnan et al., 2015; Enebe and Babalola, 2019). In addition to improving our mechanistic understandings of plant physiology, plant-microbe mutualisms and their manipulation have become the focus of research into plant husbandry with applications ranging from crop improvement to vegetation restoration (Chaparro et al., 2012; O’Callaghan, 2016; Castro et al., 2018; Compant et al., 2019).

Seagrasses are marine plants characterised by several eco-physiological traits that allow them to live submerged in water (Les et al., 1997; Hemminga and Duarte, 2000; Kuo and den Hartog, 2006). They are globally distributed throughout the coastlines of all continents (except Antarctica), and are key players in sequestration of carbon dioxide, coastal protection and support of human communities by food provision and tourism (Costanza et al., 1997; Mtwana Nordlund et al., 2016). By comparison to terrestrial plants, understanding of the nature of seagrass-bacteria interactions, and the mechanisms through which bacteria are acquired is limited. This is astonishing, given the attention that plant microbiomes have received from the past two decades, and the significant value of seagrasses ($AUD 3.9–5.4 billion for carbon sequestration; Lavery et al., 2013).

As seen in terrestrial vascular plants, distinct seagrass-associated bacterial communities inhabit discrete microenvironments within and around the plant, including the internal tissue of leaves (phyllosphere), roots (endosphere) and the layer of sediment directly influenced by the plant activity (rhizosphere) (Crump and Koch, 2008; Garcias-Bonet et al., 2012; Sun et al., 2015; Hurtado-McCormick et al., 2019). The different physico-chemical conditions within these microenvironments, such as oxygen and nutrient concentrations, are likely to favour the divergent bacterial communities (McRoy and Goering, 1974; Kilminster and Garland, 2009; Chaparro et al., 2014; Martin et al., 2019). Seagrass microbiomes are involved in many processes that benefit the plants (Ugarelli et al., 2017; Tarquinio et al., 2019), including nutrient supply (e.g., nitrogen fixation associated with seagrass leaves and roots) and detoxification from harmful compounds (e.g., hydrogen sulphides around seagrass roots; Welsh, 2000; Jensen et al., 2007; Hamisi et al., 2009). Indeed, nitrogen fixing bacteria are estimated to provide up to 50% of the nitrogen required by seagrasses (O’Donohue et al., 1991), while sulphate oxidising bacteria alleviate seagrass below ground tissues from the toxic effects of hydrogen sulphide (Brodersen et al., 2018; Martin et al., 2019).

Recently, studies on terrestrial plants have focused on the microbiomes associated with seeds, and particularly their manipulation in order to improve plant health. Since the bacterial community established within seeds will be carried by the seedling and possibly the adult plant (Hardoim et al., 2015; Truyens et al., 2015), the quality of the seed microbiome is likely to impact the future plant fitness (Berg et al., 2017). Seed-borne bacteria are localised within the integument, endosperm and embryo of seeds (Shade et al., 2017) and their manipulation has been particularly significant to reduce, for example, the incidence of plant disease (Adhikari et al., 2001; Bloemberg and Lugtenberg, 2001), increase agricultural production (O’Callaghan, 2016), improve seedling development (Compant et al., 2010; Johnston-Monje and Raizada, 2011; White et al., 2019), promote germination and induce plant defence mechanisms (Weyens et al., 2009; Sánchez-López et al., 2018).

The characteristics and origins of the seagrass seed microbiome is largely unexamined. Seagrasses can be monoecious, with flowers containing male and female organs, or dioecious, with separate male and female plants. During the reproductive season, male flowers produce a hydrophilic pollen that fertilises ovules in female flowers, which can produce up to 60 seeds, depending on the species (Orth et al., 2007). Seeds represent a crucial phase in the life cycle of flowering plants, including seagrasses, because they maintain genetic diversity within a population (Kendrick et al., 2017) and, in some species, can persist for years in a dormant state developing into a new plant only when the appropriate conditions are met (Kuo and Kirkman, 1992; Orth et al., 2000). *Halophila ovalis* is a dioecious marine plant present in tropical Indo-Pacific waters as well as Australian temperate waters (Short et al., 2007, 2010) and is a foundation species in the Swan River (Southwestern Australia) where it plays an important role in terms of sediment stability and ecology (Hillman et al., 1995). *H. ovalis* exhibits seasonal colonisation, and, as with other opportunistic species, relies mainly on sexual reproduction and seed recruitment for the annual regeneration of meadows (Statton et al., 2017). The interest in exploring *H. ovalis* seed microbiome extends beyond that of exploring a potential biodiversity niche, as seed borne bacteria are likely to impact seedling germination and development, therefore affecting meadows enduring.

When endophytic bacteria form spatially and temporally stable associations with their host we refer to them as core microbiome (Astudillo-García et al., 2017). Core microbiomes can be defined by taxonomy or functionality (i.e., metagenomic or metatranscriptomic analyses used to predict shared functions). Moreover, we can define core microbiomes at a population level (i.e., the bacteria shared between plants of the studied population) or at a species level (i.e., the bacteria shared between plants from different populations; Vandenkoornhuyse et al., 2015). In each case, the plant core microbiome is considered to contain key microbial taxa that are critical for plant health and fitness (Lemanceau et al., 2017). For seagrasses, few studies have explored the existence of a core microbiome (Hurtado-McCormick et al., 2019; Martin et al., 2019).

In this study, we characterised the diversity and role of endophytic bacteria associated with a population of the seagrass *Halophila ovalis* collected from the Swan River. In particular, we characterised the composition and role of the bacterial community for six *H. ovalis* microenvironments; leaves, roots, flowers, fruits, seeds and the rhizosphere. We also investigated the presence of a *H. ovalis* core microbiome at a population level, within the six *H. ovalis* microenvironments and followed the changes of core bacteria within the reproductive tissues.

## Materials and Methods

### Sample Collection and Processing

Seagrass samples were collected from a female *H. ovalis* meadow located at Pelican Point, Swan-Canning Estuary, in southwestern Australia (−32.026S; 115.7580E). *H. ovalis is* dioecious, with separate female and male flowers that develop during the austral summer (between December and February). Fruits start to develop from February till May and each fruit can contain up to 60 seeds (each about 2-mm diameter) which are released into the surrounding sediment once they reach maturity. Plant material was collected during two different occasions: February 2019 when the plants had immature flowers (Time 1) and April 2019, when the plants had developed mature fruits (Time 2). Flowers and mature fruits were collected while still attached to the adult plant. Leaves, roots and sediment were collected during each occasion (five replicates on each occasion) while flowers, fruits and seeds were collected once (each represented by five replicates), reflecting the reproductive phenology of *H. ovalis*.

Replicates were collected using a sterile PVC core of 10-cm diameter (1 mt depth), at a distance of 0.5 m apart; cores were stored in sterile zip-sealed plastic bags, in an ice box filled with ice until delivery to the laboratory, where they were frozen. The rhizospheric sediment was kept attached to the roots within the zip-sealed plastic bags, detached from the roots in the laboratory and kept in 2.5-ml sterile tubes at −20°C.

All the seagrass samples were processed in order to remove plant epiphytes and obtain only microbial endophytic DNA. All samples were rinsed in 0.2 μm filtered water collected from the river and, sediment-free tissues were then immerged in 70% ethanol for 30 s followed by a rinse in sterile Milli-Q water (MoBio Laboratories, Carlsbad, CA, United States). This procedure was repeated three times, with a final rinse in Milli-Q water performed three times to remove any excess of ethanol (Saldierna Guzmán et al., 2020). Fruits were surface sterilised as described above prior to opening with a sterilised razor blade in order to remove the seeds. Seeds were then placed in sterile 2.5 ml tubes and surface sterilised as described above.

### DNA Extraction and PCRs

Between 0.132 and 0.333 wet weight grams of samples were weighed for each replicate and tissue type and DNA was extracted using a PowerSoil Kit (MoBio) following the manufacturer’s instructions (on average, sediments = 0.27 g, roots = 0.25 g, leaves = 0.24 g, flowers = 0.24 g, fruits = 0.26 g, and seeds = 0.23 g). For each replicate of *H. ovalis* tissue, 15 flowers, between 8 and 13 fruits, between 120 and 180 seeds, 5 to 6 leaves and about 15 roots were pooled in order to obtain enough material for the DNA extraction. Final extracted DNA concentration was checked by using the QIAxpert machine from QIAGEN (which calculates DNA concentration of samples from the measured UV/VIS absorption spectrum) at CSIRO facility. Extracted DNA was then eluted and kept in 50 μL nuclease-free water.

PCRs were performed on extracted samples to screen for bacterial presence. GoTaq Green Master Mix (Promega) was used for the PCR reactions and primer concentration was 0.7 μM. Primer pairs used for the present study were 515F 5′ – GTGCCAGCMGCCGCGGTAA -3′ and 806R 5′ – GGACT ACHVGGGTWTCTAAT – 3′ primers which target the V4 hypervariable region of the 16S rRNA gene (Caporaso et al., 2012). PCR cycling conditions involved an initial step at 95°C during 120 s followed by 32 cycles of denaturation at 95°C for 30 s, annealing at 55°C for 30 s and extension at 72°C for 60 s; followed by a final extension step at 72°C for 10 min in accordance to the guideline of the Taq DNA furnisher’s (Promega).

### Bioinformatic Analyses

Microbial communities were sequenced by the Australian Genome Research Facility (AGRF) on the Illumina MiSeq platform, using Nextera XT v2 indices and 300 bp paired end sequencing chemistry. Bioinformatic analysis of sequence reads were performed in MOTHUR (v1.39.3; Department of Microbiology and Immunology, The University of Michigan)^1^ using the Standard Operating Procedure (SOP, page accessed on September 2019) (Schloss et al., 2009; Kozich et al., 2013). Paired end reads were assembled by aligning the forward and reverse amplicon sequence reads, which joined paired reads using the *make.contigs*() command with the option trimoverlap = T. Sequences with ambiguous bases and and/or containing a homopolymer stretch longer than eight bases were removed. Unique contigs were aligned to the SILVA database (version 132) (Cole et al., 2014) using the *align.seqs*() command with default settings. Following alignment, sequences were pre-clustered (*diffs = 2*) for further error reduction and *chimera.uchime*() command (Edgar et al., 2011) was used for de novo removal of chimeric reads. Reads were clustered using the default Opti clustering method in MOTHUR at 97% similarity to produce Operational Taxonomic Units (OTUs). A “blank” sample of reaction buffers was also sequenced and OTUs present in the control (predominantly Clostridium_XlVa) were removed from all other samples. Also, OTUs represented by a single sequence and/or not present in at least three samples were removed. The remaining OTUs were classified using the SILVA taxonomy database *method = wang* (cutoff probability value = 80; Wang et al., 2007; Quast et al., 2013) and OTUs identified as chloroplast, mitochondria or algae (12.4% of total sequences) were removed. The most abundant unclassified OTUs were using megablast against the nucleotide database (nr/nt) using the NBCI (National Center for Biotechnology Information, U.S. National Library of Medicine) web portal^2^ to assign them a taxonomy using the best blast hit.

### Statistical Analyses

Data visualisation and statistical analyses were performed in R (version 4.0.2) (R Core Team, 2020) using the phyloseq (McMurdie and Holmes, 2013), ggplot2 (Wickham, 2016) and vegan (v 2.5–6; Oksanen et al., 2019) packages. Considering the number of reads (or number of sequences) per sample for a specific taxon as a proxy of its abundance (Calle, 2019), OTUs with a relative abundance across the full dataset of <1% were removed from the analyses to eliminate the influence of possible sequencing artefacts. A final number of 710 taxa (OTUs) from the six seagrass microenvironments (i.e., five types of tissue – leaf, root, flower, fruit, seed – and sediment) were kept for downstream analyses.

Bacterial diversity within samples (alpha diversity) was estimated using richness (number of observed OTUs) and Shannon’s diversity (Shannon and Weaver, 1964). Analysis of Variance (ANOVA) was performed to test hypotheses about differences in diversity between seagrass microenvironments and sampling occasions. A Tukey’s Honest Significant Difference (HSD) test was used (Tukey, 1953; Kramer, 1956, 1957) to identify which pairwise comparisons were significant.

Principal coordinate analysis (PCoA) was used to visualise patterns in the microbial assemblage (i.e., beta diversity) among the seagrass microenvironments, based on Bray-Curtis dissimilarities (Bray and Curtis, 1957) calculated from log10-transformed OTU abundances. To test hypotheses about differences in the bacterial assemblage among microenvironments, a permutational multivariate analysis of variance (PERMANOVA) was performed using the *adonis* function from the *vegan* package in R with 999 permutations (Anderson, 2017).

To explore which taxa most contributed to patterns, we used similarity percentage analysis (SIMPER, 999 permutations, log10 +1 transformed abundance; Clarke, 1993) to. The first two OTUs with the highest contribution to the dissimilarity between pairwise tests were extracted and fitted onto the PCoA with the function *envfit* from the package *vegan* (Oksanen et al., 2019).

To explore the possible role of bacteria within the seagrass holobiont, we were able to assign a function to 247 taxa based on their family or species taxonomic identity using the database FAPROTAX (Louca et al., 2016) and literature search (Supplementary Table 1). The two major limitations in applying FAPROTAX or other trait databases, are (1) databases are limited/incomplete in terms of their ability to capture all organisms. They are biased toward more heavily studied and cultured organisms, and (2) the assumption that if cultured bacterial species are able to perform a particular function, then all members of the same taxon also share and exhibit this phenotype. Despite these limitations, we were able to identify in this analysis bacteria involved in nitrogen cycling (nitrogen fixing, nitrifying, and denitrifying bacteria), sulphur cycling (sulphide-oxidising and sulphur-reducing bacteria) and/or with known plant growth promoting properties (PGPB: bacteria able to produce phytohormones).

Stable associations between *H. ovalis* and specific taxa were investigated through an exploratory analysis of the core microbiome. OTUs present (relative abundance >0%) in all samples of each microenvironment were considered part of their core microbiome. Moreover, we tested for the presence of a seagrass core microbiome present across samples of vegetative tissues (i.e., leaves and roots) and among reproductive parts (flowers, fruits and seeds), the latter to show which OTUs were conserved from the flower to the seed stage.

## Results and Discussion

### Components of the Bacterial Community

**FIGURE 1:**
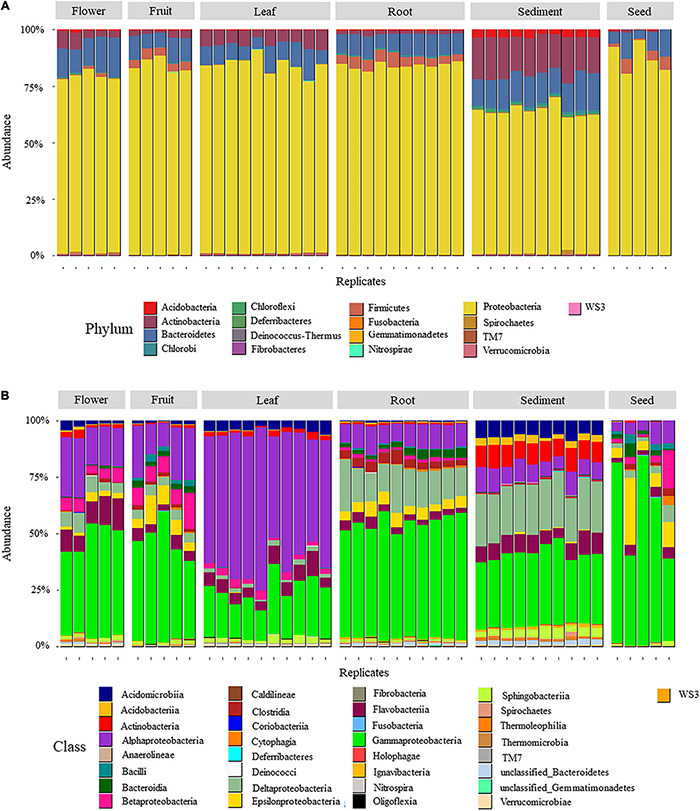
Relative abundance of OTUs aggregated by phylum **(A)** and class **(B)** in six *H. ovalis* microenvironments (rhizospheric sediment, roots, leaves, flowers, fruits, and seed). Numbers are presented in percentage.

Seven hundred and ten OTUs were identified from high quality 16S rRNA gene sequences of the 45 samples. The majority of these OTUs (426 out 710, Figure 1A) belonged to the phylum Proteobacteria which was the dominant lineage within all six microenvironments, representing 87%, 84%, 83%, 83%, 78%, and 63% of the OTUs within seed, fruit, leaf, root, flower, and sediment, respectively (Supplementary Table 2). Gamma-, Delta-, and Alpha-proteobacteria were the dominant proteobacterial classes (97, 131, and 173 OTUs, respectively), compared to Oligoflexia and Beta- and Epsilon-proteobacteria (3, 9, and 13 OTUs, respectively).

With 140 unique OTUs, the Bacteroides phylum contained the second highest number of OTUs associated with *H. ovalis* microenvironments (Figure 1A and Supplementary Table 2). Bacteroides represented from 8 to 14% of the bacterial community in different microenvironments. Flavobacteriia (phylum Bacteroides) was the most relatively abundant Bacteroides class, being relatively prevalent in flowers (11%), sediment (8%) and leaves (6%, Figure 1B), while Sphingobacteriia, the second most abundant Bacteroides class, was most relatively abundant in sediment (3.5%). The remaining phyla were less relatively abundant, but they showed a clear association with specific seagrass microenvironments. For example, Acidobacteria (31 unique OTUs) and Actinobacteria (46 unique OTUs) were mainly present in the rhizosphere and almost absent in seagrass tissues (Figure 1B).

### Differences in Diversity and Richness Between Microenvironments

Alpha diversity (based on a total number of 1565233 sequences, Supplementary Figure 1) measured as Shannon’s diversity index and observed OTUs, varied significantly between seagrass microenvironments (ANOVA, *p*_*observed*_ < 0.0001, MS_*observed*_ = 3.7, *F*_*observed*_ = 27.7; *p*_*shannon*_ < 0.0001, MS_*shannon*_ = 71418, *F*_*shannon*_ = 34.6). However, alpha diversity did not vary substantially among the two sampling occasions (ANOVA, *p*_*observed*_ = 0.8375, *p*_*shannon*_ = 0.8752) and so this was excluded from further statistical analyses. In general, diversity and richness within seeds was lower than all other microenvironments. Sediment was characterised by the highest diversity and richness among microenvironments and was significantly different from *H. ovalis* tissues, except for fruits and flowers (Supplementary Figure 1 and Supplementary Table 3). Bacterial community diversity and richness of roots, flowers, leaves and fruits were not significantly different although these parameters tended to be smaller for the leaf bacterial community.

### Microenvironment-Specific *Halophila ovalis* Microbiomes

**FIGURE 2:**
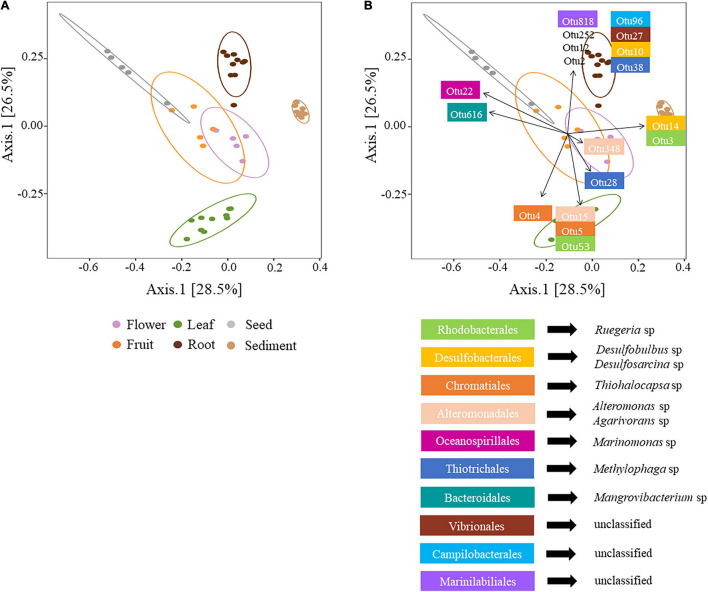
**(A)** Principal coordinates analysis (PCoA) of microbial communities associated with *H. ovalis* rhizospheric sediment, roots, leaves, flowers, fruits, and seeds. The percentages in parentheses refer to the proportions of variation explained by each ordination axis. The confidence ellipses (with a 95% confidence level) corresponding to each microenvironment are shown **(B)** PCoA with envfit object displaying the first two OTUs with the highest contribution to the dissimilarity between pairwise tests from SIMPER. OTUs identity is reported below the PCoA. OTU 2, 12, and 252 are unclassified Gammaproteobacteria.

The first two axes of a PCoA comparison of bacterial diversity explaining 55% of bacterial variability among microenvironments. Clear partitioning of the communities belonging to the leaves, roots and sediment and a less accentuated separation for flowers, fruits and seeds was evident (Figure 2A). These patterns of differences in bacterial community structure according to the plant microenvironment was also supported by PERMANOVA, (*p* < 0.0001, Supplementary Table 4), where all bacterial communities were significantly different from each other. The different physical and chemical conditions within discrete seagrass microenvironments are likely to favour microscale heterogeneity in bacterial diversity and community structure (Borum et al., 2007; Ettinger et al., 2017; Brodersen et al., 2018; Hurtado-McCormick et al., 2019).

### A Distinctive Rhizosphere Community

The bacterial assemblage inhabiting *H. ovalis* rhizosphere represented a clearly distinguished cluster from the other microenvironments (Figure 2A). The bacterial community found in this microenvironment was, on average, the richer (400.6 ± 6.56 total number of OTUs) and more diverse than those associated with *H. ovalis* tissues. This is not surprising since in terrestrial systems, the rhizosphere is characterised by an increased diversity compared to host-associated communities (Edwards et al., 2015) and our study is consistent with many studies of seagrass microbial diversity (Jensen et al., 2007; Mejia et al., 2016; Fahimipour et al., 2017; Martin et al., 2018, 2020).

The main driver of the clustering of the rhizospheric bacterial community was the high abundance of Desulfobacterales (especially Desulfosarcina sp.) and Chromatiales (*Chromatiaceae*) which accounted for 17%, and 17.5% of rhizospheric sequences, respectively (Figure 2B and Supplementary Table 5). Members of these two taxa have been shown to dominate the rhizosphere of seagrasses (Sun et al., 2015; Ettinger et al., 2017; Cúcio et al., 2018) and salt marsh plants (Thomas et al., 2014), where they influence sulphur and nitrogen cycling (Cúcio et al., 2016). Actinobacteria was the third most relatively abundant phylum in sediment (17%), represented by the classes Actinobacteria (9%) and Acidimicrobiia (7%), while its average abundance was less than 6.5% of sequences within *H. ovalis* tissues. In general, within the oxygenic sediment layer and/or at the root-sediment interface, members of the Actinobacteria, Flavobacteriales, and Myxococcales, can play a prominent role in degrading organic substrates (Lu et al., 2006; Bowman et al., 2012; Větrovský et al., 2014; Lasa et al., 2019). For example, bacteria belonging to the genera *Eudoraea* and *Maribacter* (Flavobacteriaceae) found in the present study, can grow aerobically utilising several organic substrates (e.g., organic acids, amino acids, and carbohydrates; Alain et al., 2008; Handley and Lloyd, 2013). On the contrary, within the anoxic layers of seagrass sediment, decomposing processes can be based on the activity of obligate fermentative *Clostridium* species, which provide substrates to other organisms, such as sulphate-reducing bacteria (SRB; Küsel et al., 1999).

Sulphate reduction is one of the main processes in marine sediments related to the anoxic mineralisation of organic matter (Jørgensen, 1982) and it is reported as high in seagrass meadows, where it is fuelled by organic exudates from the plant roots (Isaksen and Finster, 1996; Holmer and Nielsen, 1997; Hansen et al., 2000; Cúcio et al., 2016). This process it also likely to be significant in eutrophic environments such as the Swan-Canning Estuary, where sediments have been characterised by nutrient and organic matter enrichment (Kelsey and Western Australia, Department of Water, 2010). SRB gain energy for cell synthesis and growth by coupling the oxidation of organic compounds to the reduction of different sulphate/sulphur molecules to hydrogen sulphide (H_2_S, HS^–^; Dworkin et al., 2006; Camacho, 2009). *H. ovalis* rhizospheric sediment contained a diverse assemblage of OTUs belonging to Desulfobacterales, Desulfovibrionales and Desulfuromonadales known to reduce elemental sulphur to H_2_S (Muyzer and Stams, 2008; Varon-Lopez et al., 2014). Among them *Desulfosarcina* sp. was associated only with the sediment community in our study (Figure 2B). However, sulphides that are produced from the reduction of sulphur, in particular H_2_S, are toxic to plants (Koch and Erskine, 2001; Borum et al., 2005; Lamers et al., 2013). Several studies have described that the association of seagrass below-ground parts with sulphide-oxidising bacteria (SOB) can release the marine plants from sulphide toxic stress (van der Heide et al., 2012; Fahimipour et al., 2017; Martin et al., 2019; Van Der Geest et al., 2020). In fact, SOB by using H_2_S for their metabolism demand could help to reduce sulphide concentration, as suggested for *Zostera* species (Hansen et al., 2000; Nielsen et al., 2001; van der Heide et al., 2012; Brodersen et al., 2018). In our study, four rhizospheric OTUs (relative abundance of 0.15) were putatively involved in the oxidisation of sulphides such as *Rhodovulum* sp., (Rhodobacterales) and *Thiohalocapsa* sp. (Chromatiales) which is able to oxidize both sulphide and elemental sulphur (Straub et al., 1999; Hamilton et al., 2014).

Microbially mediated denitrification is a key ecosystem service provided by seagrass associated bacteria (Reynolds et al., 2016), which acts to remove the excess of nitrogen from eutrophic systems (Stohr and Ullrich, 2002). This may be especially important in estuarine systems which usually present high concentrations of nutrients. For example, *Caldithrix* sp. (Deferribacterales) is an anaerobic nitrate reducer, and *Saccharospirillum* sp. (Oceanospirillales) is able to catalyse the three steps of the denitrification process (Miroshnichenko et al., 2003; Zhang et al., 2019). OTUs affiliated with *Ignavibacterium* sp. were mostly present in sediment samples; *Ignavibacterium* species are able to reduce ammonium to dinitrogen gas (N_2_) that could be used by nitrogen fixing bacteria present within the seagrass ecosystem. While organic nitrogen recycling in the rhizospheric sediments can provide a large proportion of the plant’s nitrogen demand, it may be insufficient to meet the annual N requirement of the plant, due to the limited nutrient diffusion and root uptake rates (Lepoint et al., 2002). Nitrogen fixation might represent another important source of nitrogen used by seagrasses and high rates of nitrogen fixation have been recorded in sediments colonised by diverse seagrass species (Welsh, 2000; Bagwell et al., 2002). Some members of Desulfobulbaceae and Desulfobulbacteraceae could be involved in anaerobic nitrogen fixation (Herbert, 1999; Sun et al., 2015) as well as *Cohaesibacter* sp. which has already been found associated with *H. ovalis* roots in the Swan River Estuary (Martin et al., 2020). Finally, *Nitrosococcus* sp. (Chromatiales), oxidises nitrites (Bock and Wagner, 2013), which are toxic to plant tissues, to nitrates which can be up taken by seagrass roots and translocated to leaves for storage in vacuoles (Touchette and Burkholder, 2000).

### Halophila Ovalis Root Microbiome

Bacterial assemblages characterised from roots were distinct from those of other microenvironments (Figure 2A). Nonetheless, bacterial diversity was comparable to that of reproductive tissues and leaves, consistent with results found for *Zostera marina* (Ettinger et al., 2017). The root bacterial community mainly constituted Proteobacteria, in which Gamma- and Deltaproteobacteria were highly abundant (52% and 15.5% of the sequences, respectively). Thiotrichales, Vibrionales (Gammaproteobacteria), Campylobacterales (Epsilonproteobacteria), and Clostridiales (Firmicutes), were highly abundant in roots and reproductive tissues, but absent or almost absent in leaves and sediment (Figure 2B). Desulfobacterales were abundant in roots and sediment, but not in other tissues while Fibrobacterales were only present within the roots.

A number of studies have shown that the order Clostridiales is often part of the microbiome of seagrass species (e.g., *Z. marina*, *Halodule wrightii;* Küsel et al., 1999; Cúcio et al., 2016; Garcias-Bonet et al., 2020). This association between Clostridiales and seagrasses could be a potential aid for seagrass health, since some members of this order can be involved in nitrogen fixation (Minamisawa et al., 2004), fermentation (Cato et al., 1986), as well as sulphate reduction (Widdel, 2006). As for the sediment community, an ability to reduce sulphate and sulphur was a common feature of a high proportion of root-associated bacteria, in particular Desulfobacterales (13.5%; Supplementary Table 5). Interestingly, some genera within the family Desulfobacteraceae are also able to oxidize alcohols. During the night cycle, the lack of oxygen around the roots leads seagrass root tissues to switch to fermentation, which causes the release of ethanol to the rhizosphere (Smith et al., 1988). Cúcio et al. (2016) suggested that this association might represent a fair trade between host and microbes, where bacteria use ethanol as an electron donor at night and remove the alcohol from the surrounding of the roots (Kuever et al., 2005).

However, reduction of sulphur and sulphate may be toxic to plants and, in the case of seagrass roots, Campylobacterales may serve as detoxicant. In the present study, the families Campylobacteraceae and Helicobacteraceae were part of the root community (nine OTUs with a relative abundance of 5%; Figure 2B). *Helicobacter* sp. as well as *Arcobacter* sp. and *Sulfurimonas* sp. are important SOB commonly found within below-ground parts of several seagrasses (i.e., *Z. marina* and *H. ovalis*, Küsel et al., 2006; Jensen et al., 2007; Lu et al., 2020; Martin et al., 2020); the high relative abundance of *Helicobacter* sp. OTUs contributed to the distinct clustering of the root community. Moreover, some SOB may be involved in the recycling of nitrogen in seagrass beds. For example, one OTU from our study matched at 99% with *Arcobacter nitrofigilis*, a nitrogen fixing bacterium isolated from the roots of the salt marsh *Spartina alterniflora* (McClung et al., 1983). Likewise, some *Sulfurimonas* species are able to use nitrate and/or nitrite as electron acceptors (Han and Perner, 2014). Other SOB associated with *H. ovalis* roots belonged to Thiotrichales and were relatively abundant (5.5%). Thiotrichales are filamentous sulphur oxidising bacteria (Garrity et al., 2005) which have been found abundant in the rhizosphere of seagrasses from Portugal (Cúcio et al., 2016) and *Z. marina* leaves (Ettinger et al., 2017).

Operational Taxonomic Units from Vibrionales that were present in the roots (6%) were almost completely absent in the sediment and phyllosphere (0.5 and 0.6%, respectively) and contributed to the clustering of the root community (Figure 2B). Members of the Vibrionales have been found within seagrass sediments (*Enhalus acoroide* and *Thalassia hemprichii*; Liu et al., 2018) or associated with seagrass roots (Martin et al., 2020). Although the role of Vibrionales within the seagrass microbiome is not clear and our OTUs may be simply related to potential pathogenic species (e.g., *Vibrio* sp.; Blazer et al., 1988; Liu et al., 2018), some members might be involved in nitrous oxide (N_2_O) reduction (Hanke et al., 2016). Bacteria from the Fibrobacterales (*Fibrobacter* sp.) which have been found associated with the roots of *H. wrightii*, but not with the surrounding sediment (Stuij, 2018), were also only found in root samples in the present study. They are cellulolytic bacteria that can degrade lignocellulose and this capability has been hypothesised to be an essential feature related to the ability of those bacteria to colonise plant roots (Jose et al., 2014; Kandel et al., 2017; Liu et al., 2017).

### *Halophila ovalis* Leaf Microbiome

The bacterial community in *H. ovalis* leaves was significantly different from the microbiomes associated with the rhizospheric sediment and seagrass reproductive tissues (Figure 2A). Alphaproteobacteria were the most abundant bacterial phylum in leaf samples. Some of these, such as Caulobacterales and Sphingomonadales, were almost absent from the other microenvironments, while others were present more generally in the plant (e.g., Rhodobacterales; Figure 2B and Supplementary Table 5).

The family Rhodobacteraceae (Rhodobacterales) has been associated with the phyllosphere of several seagrass species (*Z. muelleri* and *Thalassia hemprichii*; Jiang et al., 2015; Hurtado-McCormick et al., 2019; Rotini et al., 2020) and comprises photosynthetic purple non-sulphur bacteria which are primary surface colonizers in marine habitats (Palacios and Newton, 2005; Dang et al., 2008). Interestingly, the ability of colonising and growing on surfaces may be linked to their capacity to produce antibacterial compounds which could prevent other bacteria/pathogens from colonising the surface and/or form a biofilm, therefore protecting their host (Dang et al., 2008). Some members of Rhodobacterales are also able to denitrify. For example, *Labrenzia* sp. found in the present study, which has been previously isolated among epiphytes of *P. oceanica* leaves and endophytes of *H. ovalis* roots, is involved in conversion of N_2_O to N_2_ (Blanchet et al., 2017; Martin et al., 2020). Species of *Labrenzia* are also known to be plant growth promoters in terrestrial environments because they can produce auxin which might be of a particular importance for supporting plant growth (Liu et al., 2014; Fidalgo et al., 2016).

Other bacteria involved in nitrogen cycling were present within the leaf endophyte community. For example, *Filomicrobium* sp. (Rhizobiales) is able to utilize organic molecules (e.g., ammonia) as the N source for growth (Wu et al., 2009). Flavobacteriales (phylum Bacteroidetes) such as *Muricauda* sp. *Aquimarina* sp. and *Maribacter* sp. may detoxify seagrasses from N_2_O through its reduction to N_2_ (Xu et al., 2015; Nakagawa et al., 2019). Finally, five OTUs belonged to Sphingomonadales and Caulobacterales. Among them, one OTU matching with *Altererythrobacter* sp. is able to resist metal contamination (Wu et al., 2014), a trait that could be useful in the phyllosphere of *H. ovalis* since high metal concentrations in Swan-Canning River have been detected (Kelsey and Western Australia, Department of Water, 2010).

Alteromonadales and Chromatiales (Gammaproteobacteria) were also relatively abundant within leaves compared to the other seagrass tissues and seemed to be of particular importance for the clustering of leaf community in our study (Figure 2B). *Granulosicoccus* sp. and *Ruegeria* sp., harbour many genes involved in sulphur metabolism comprising a gene for dimethylsulfoniopropionate (DMSP) demethylase (Kang et al., 2018; Wirth et al., 2020). DMSP can be produced by macroalgae and higher plants as osmoregulator, herbivore deterrent and antioxidant against reactive oxygen species (Jonkers et al., 2000; Husband et al., 2012) and represent an important source of reduced carbon and sulphur for marine bacteria (Wirth et al., 2020). Genetic evidence indicates that several bacteria commonly found within seagrass leaves share the capacity to utilise DMSP that may represent a trait characteristic of seagrass leaf associated bacteria (Crump and Koch, 2008; Lucas-Elío et al., 2012).

### Flower and Fruit Microbiomes

Flower and fruit communities were mainly composed by bacteria from the phyla Proteobacteria and Bacteroidetes. Although fundamental knowledge of flower-associated microbiota remains largely unknown, when Shade et al. (2013) studied the microbiome of apple flowers they found diverse taxa affiliated to Proteobacteria, Actinobacteria, and Bacteroidetes. In the present study, the Proteobacterial families *Piscirickettsiaceae* (*Methylophaga sp.*), *Campylobacteraceae*, *Helicobacteraceae*, and *Methylophilaceae* were highly represented in these tissues. *Methylophaga* species have been isolated from diverse marine environments (Janvier et al., 1985; Doronina et al., 2007) where they are likely involved in denitrification processes (Auclair et al., 2010). Also, nitrogen fixation is likely to occur in those communities, since Campylobacterales were identified as nitrogen fixers when isolated from *Spartina* sp. roots (McClung and Patriquin, 1980). *Marinomonas* sp. (*Oceanospirillaceae*), a plant growth promoting bacteria found associated with flower and fruit microbiomes is known to play a key role in *P. oceanica* seedling growth (Celdrán et al., 2012). *Alteromonas* sp., associated with the flower community, but not the fruits, can have algicidal proprieties (Sakami et al., 2017) and associations between seagrass and algicidal bacteria have already been reported (Inaba et al., 2017). Finally, Diskin et al. (2017) reported the family *Chitinophagaceae* (Bacteroidetes) to be the most abundant one within the mango fruit microbiome. Some species from this family degrade chitin, while others hydrolyse cellulose (Rosenberg, 2014). In our study one OTU belonged to *Chitinophagaceae* and it was found only in fruits and seeds but not in flowers.

Bacteria belonging to Deinococcus-Thermus and TM7 phyla were also members of the apple flower microbiome, although they were not as abundant as in the present study. This discrepancy may be link to a different abundance of those phyla related to the age of flowers. For example, TM7 taxa are contenders for colonisation of closed flowers, where they survive but do not grow until flowers open and then grow rapidly and competitively on open flowers (Shade et al., 2013). Interestingly, *Truepera* sp. (Deinococcus-Thermus) found in this study, is also associated with apple flowers (Shade et al., 2013), which may suggest a possible important role within the flower microbiome. *Truepera* sp. seems to possess important adaptations to different environmental stress, such as resistance to desiccation, ultraviolet radiation, high salinity, and high temperature (Blasius et al., 2008), which can be experience in the Swan River during summer time when *H. ovalis* meadows became exposed to air and water temperatures can raise up to 30°C in the hottest months (Kelsey and Western Australia, Department of Water, 2010).

### Seed Microbiome

Seed endophytes have intensively been study in terrestrial plants, in particular crops (Johnston-Monje and Raizada, 2011; Adam et al., 2018; Chen et al., 2020), since they can assist seedling germination and cope with environmental stresses, such as drought or salinity (Truyens et al., 2015; Nelson, 2018). *H. ovalis* seeds were characterised by the lowest number of OTUs compared to the other microenvironments, and were mainly composed of Gammaproteobacteria. A similar pattern in terms of OTU abundance and diversity has been found for the seeds of the pumpkin *Cucurbita pepo* (Adam et al., 2018) and the spinach *Spinacia oleracea* (Lopez-Velasco et al., 2013). Three OTU belonged to the genus *Marinomonas* (Figure 2B and Supplementary Table 5); *M. posidonica* may enhance the growth of *P. oceanica* seedlings, induce changes in the epiphytic bacterial community and have a regulatory effect on macro-epiphyte structure (Celdrán et al., 2012). Two OTUs were related to *M. communis* which exhibits grow-promoting traits when found in plants (Mesa et al., 2015). Another OTU belonged to the genus *Mangrovibacterium* (Bacteroidales), a nitrogen-fixing bacterium isolated previously from mangrove sediment (Huang et al., 2014). Moreover, members of the family *Pseudoalteromonodaceae* were more abundant in the seed bacterial community compared to other tissues. Species of the genus *Pseudoalteromonas* are common within the marine environment and have been found associated with corals, sponges, seagrass surface and within molluscs (Romanenko et al., 2008). *P. luteoviolacea* (found in this study) is a globally distributed marine bacterium that can induce the metamorphosis of tubeworm and coral larvae (Alker et al., 2020) Finally, one OTU matching with *Methylophaga thiooxydans* was found in the present study within the seed microenvironments. Methanol-consuming bacteria are common in marine environments and include members of the genera *Methylophaga* and *Methylobacter*. Angiosperms, and so seagrasses, produce methanol as a by-product of cell-wall synthesis (Nemecek-Marshall et al., 1995), yet it has been reported that methanol could inhibit germination and/or negatively affect the growth of angiosperm seedlings (Abanda-Nkpwatt et al., 2006). In strawberry plants, this negative effect may be mitigated by methanol-consuming bacteria (e.g., *Methylobacterium extorquens;* Abanda-Nkpwatt et al., 2006; Kurilenko et al., 2010). Moreover, *Methylophaga* species isolated from rhizospheric sediments are also capable of producing auxins and therefore may be able to boost the seedling growth. However, more studies related to seagrass seed-borne bacteria are needed to understand how seagrass seed acquire bacteria, the diversity of seed-borne bacteria and potential role in seedling germination and growth.

### Core Microbiome

**FIGURE 3:**
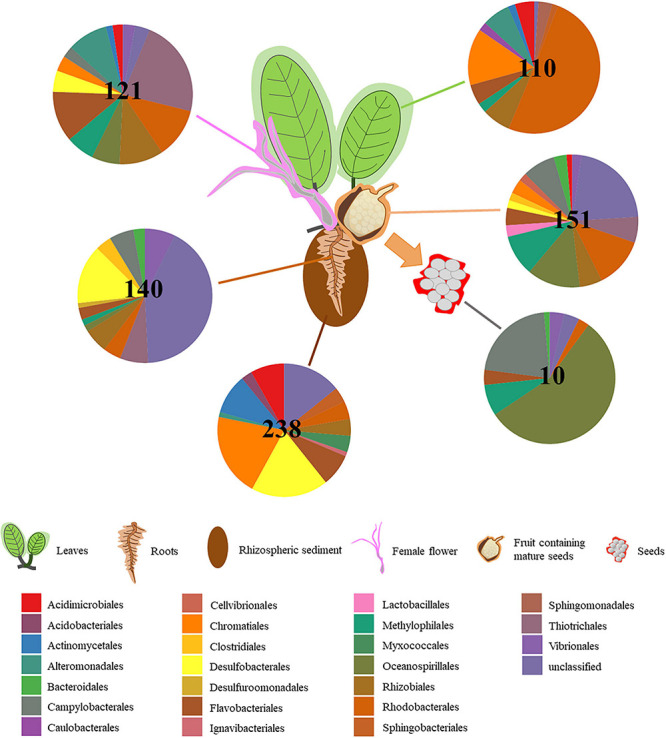
Core microbiome composition varies depending on *H. ovalis* microenvironment. Pie charts show the relative abundances of the major bacterial orders that are detected within the core microbiome of the rhizospheric sediment, rhizosphere, root, leaf, flower, fruit, and seed. Only orders that represented >0.1% of the total population are included. In the centre of the pie charts is reported the number of OTUs present in the core microbiome of each microenvironment.

The number of taxa in core microbiomes varied substantially between microenvironments, ranging from 10 OTUs for seeds, up to 238 OTUs for rhizospheric sediment (Figure 3). Although our criterion was quite strict (i.e., OTUs present in 100% of samples from a microenvironment), the number of OTUs was high relative to those found in *Zostera muelleri* (i.e., 102 OTUs in sediment, 61 OTUs in roots, two OTUs in leaves; Hurtado-McCormick et al., 2019). However, it needs to be taken in consideration that previously works have investigated seagrass core microbiomes at a species level rather than population, thus environmental variability across sampling sites could reflect the high variability of bacterial microbiomes. For example, Martin et al. (2020) found out that the root microbiomes of *H. ovalis* plants sampled in different locations within the Swan River presented a greater divergence of microbial communities than populations sampled around the Leschenault Peninsula. For the present study, the core microbiome of the six microenvironments included 63 families from 44 bacterial orders, across 10 phyla (Supplementary Table 6). Only one OTU, belonging to the family Methylophilaceae, was observed in all 45 samples, indicating that the seagrass microenvironments represent markedly different microbial niches. Our results provide evidence of a clear differentiation of core bacterial communities across the different microenvironments within the seagrass, instead of a unified seagrass core microbiome. The existence of discrete core microbiomes across the different *H. ovalis* microenvironments is consistent with patterns found in terrestrial plants, for which the rhizosphere, the phyllosphere and the root endospheres host communities that are both distinct from each other and the surrounding soils (Coleman-Derr et al., 2016; Fonseca-García et al., 2016; Maggini et al., 2019). The patterns observed here are also consistent with other benthic marine organisms including corals, where distinct microbial communities colonize different microenvironments within the coral colony, coral polyps and coral tissue (Sweet et al., 2011).

The rhizospheric sediment core microbiome (238 OTUs; Figure 3) displayed the greatest abundance of OTUs. The most abundant order was represented by Chromatiales (19%), although a relatively high number of OTUs was unclassified (12%). Desulfobacterales was the second most abundant (17.5%) order present in the sediment core microbiome and it was primarily composed of *Desulfobacteraceae* (54%) and *Desulfobulbaceae* (46%).

One hundred and forty OTUs were present across all ten root replicates and were mainly related to Desulfobacterales (13.5%, comprising Desulfobacteraceae and Desulfobulbaceae), and Chromatiales. Likewise, Gamma- and Deltaproteobacteria have previously been found to be among the most abundant members of the core microbiome of below-ground structures in the seagrass *H. stipulacea* (Mejia et al., 2016) and several others seagrasses (Cúcio et al., 2016). 96 OTUs were shared between all the root and sediment replicates and belonged mainly to Desulfobacterales, followed by Acidimicrobiales, Flavobacteriales, and Rhodobacterales.

Those core microbiomes are likely to be involved in sulphur processes and nitrogen cycle in the core seagrass rhizobiome that influence the decomposition of organic material and ultimately the health of the host (Varon-Lopez et al., 2014; Lehnen et al., 2016).

The leaf core microbiome mainly consisted of Alpha- and Gamma- Proteobacteria (Figure 3), which is also consistent with previous studies (Hurtado-McCormick et al., 2019). Almost half of the 110 OTUs constituting the leaf core microbiome belonged to the Rhodobacteraceae (Rhodobacterales, 49%). The Chromatiales was second most represented in the *H. ovalis* leaf core microbiome (13.5%) and the family Chromatiaceae (11.5%) was the most abundant within Chromatiales. Members of the Rhodobacteraceae, and Chromatiaceae may favour inhabiting the *H. ovalis* phyllosphere, where they exploit the oxic conditions and high levels of dissolved organic carbon on the leaf surface (McRoy and Goering, 1974; Rotini et al., 2017; Trevathan-Tackett et al., 2020).

121 OTUs comprised the flower core microbiome and belonged mainly to the Piscirickettsiaceae (Thiotrichales, 21.5%). Rhodobacteraceae (Rhodobacterales, 11%) and Flavobacteriaceae (Flavobacteriales, 11%) were the two other families abundant in the flower core microbiome (Supplementary Table 6). As discussed above, they could be involved in nitrogen cycle (Jones et al., 2008; Nakagawa et al., 2019) and Rhodobacteraceae has been described as colonizers in marine habitats (Palacios and Newton, 2005; Dang et al., 2008). Although, fruits and flowers were likely to have similarities in bacterial community composition, the fruit core microbiome showed a high abundance of unclassified Gammaproteobacteria as well as Oceanospirillaceae, which contain plant growth promoting bacteria and Rhodobacteraceae.

The fruit core microbiome (151 OTUs) comprised bacteria belonging to Oceanospirillaceae (Oceanospirillales, 12.5%), Rhodobacteraceae (Rhodobacterales, 12%) and Methylophilaceae (Methylophilales, 9%). However, the most abundant bacteria were unclassified Gammaproteobacteria (20.5%) to which we were unable to assign taxonomy even after blasting and literature searching.

Finally, the 10 OTUs found in the seed core microbiome belonged mainly to Oceanospirillaceae (Oceanospirillales, 55.5%) and Helicobacteraceae (Campylobacterales, 17.5%; Figure 3). Interestingly, the seed core microbiome was composed of OTUs belonging to the genera *Marinomonas* and *Mangrovibacterium*. Moreover, one OTU belonging to *Labrenzia* sp. was found across the five replicates from seed microenvironment and is known to have plant growth promoting activities. The persistence of the association between endophytic bacteria that display plant growth promoting traits and *H. ovalis* seeds highlight the potential critical function performed by those bacteria in seedling development (Puente et al., 2009; Verma et al., 2017).

Samples of all three reproductive microenvironments combined (i.e., flowers, fruits, and seeds) contained 7 OTUs. This suggests the possibility that these taxa might be passed from the flowers through fruits and into seeds. Three of these OTUs were unclassified and four belonged to *Labrenzia* sp., *Marinomonas* sp., *Amphritea* sp. and *Helicobacter* sp. Excluding seeds (which contained the fewest taxa), flower and fruit core microbiomes shared 70 OTUs. The existence of complex mechanisms of bacterial transmission from flower to fruit and then to seeds appears likely and warrants further investigation. Recent studies from the terrestrial environment have shown that endophytic bacteria associated with flowers may colonize developing ovules and ultimately end up in fruits and seeds. Compant et al. (2011) demonstrated that several *Pseudomonas* and *Bacillus* species that were present in flowers and inside the xylem vessels of ovaries were also present within seeds. Furthermore, the inoculation of flowers with known bacteria resulted in significant levels of those strains within seeds (Dutta et al., 2014; Mitter et al., 2017).

## Conclusion

Microbial assemblages in six discrete microenvironments associated with *Halophila ovalis* plants were distinct and the taxa present within the microenvironments were related to those that are likely involved in ecologically important processes that benefit plants. Although the specific mechanisms of interaction between bacterial endophytes and the host remain unknown in seagrasses, bacteria previously associated with plant growth promoting characteristics (e.g., ability to produce auxin and cytoxin), were mainly found within reproductive tissues. Seagrass seed-borne bacteria that exhibit growth promoting traits, the ability to fix nitrogen and anti-pathogenic potential activity, may play a pivotal role in seed survival, as is common for terrestrial plants. Therefore, our findings are consistent with research performed on terrestrial plants and highlight the potential beneficial role of seagrass associated bacteria.

## Data Availability Statement

The datasets presented in this study can be found in online repositories. The names of the repository/repositories and accession number(s) can be found below: https://doi.org/10.25919/9dga-ra29.

## Author Contributions

FT conducted the field work together with DNA extraction, sequencing, downstream analyses, and led the manuscript writing. OA conducted statistical analyses and contributed to the literature search on bacterial putative function. MV, OB, and AB provided critical input for data interpretation. All the authors contributed to the manuscript production.

## Conflict of Interest

The authors declare that the research was conducted in the absence of any commercial or financial relationships that could be construed as a potential conflict of interest.

## Publisher’s Note

All claims expressed in this article are solely those of the authors and do not necessarily represent those of their affiliated organizations, or those of the publisher, the editors and the reviewers. Any product that may be evaluated in this article, or claim that may be made by its manufacturer, is not guaranteed or endorsed by the publisher.

## Footnotes

1
http://www.mothur.org/

2
https://blast.ncbi.nlm.nih.gov/Blast.cgi
