# Supplementary material for: Distinct Endophytic Bacterial Communities Inhabiting Seagrass Seeds
Source: Front Microbiol. 2021 Sep 21;12:703014. doi: 10.3389/fmicb.2021.703014 (PMC8491609; doi:10.3389/fmicb.2021.703014)
Supplement: Supplementary file 1 [file Table_1.DOCX]

**Supplementary Table1**. Putative role of bacteria assigned to 247 taxa based on their taxonomic identity using both literature search and the database FAPROTAX.

| LITERATURE SEARCH |  |  |
| --- | --- | --- |
| **Biogeochemical process** | **Taxonomy** | **Refence** |
| Three steps of the denitrification | Saccharospirillum | Zhou et al., 2019 |
|  |  |  |
| Nitrate reduction to nitrite | Caldithrix | Miroshnichenko, et al., 2003 |
|  | Ignavibacterium | Liu et al., 2012 |
| Nitric oxide reduction | Ignavibacterium | Liu et al., 2012 |
|  |  |  |
| Nitrous oxide reduction to dinitrogen gas | Labrenzia | Coates et al., 2017 |
|  | Aquimarina | Nakagawa et al., 2018 |
|  | Muricauda | // |
|  | Maribacter | // |
|  | Ignavibacterium | Liu et al., 2012 |
| Nitrite oxidization | Nitrosococcus | Prosser, 2005 |
| Nitrogen fixation | Arcobacter | McClung et al., 1983 |
|  | Clostridiales | Minamisawa et al., 2004 |
|  | Cohaesibacter  Mangrovibacterium | Rua and Thompson, 2014  Huang et al., 2014 |
| Oxidization of sulphides | Rhodovulum | Straub et al., 199 |
|  | Thiohalocapsa  Arcobacter | Kumar et al., 2009  Roalkvam et al., 2015. |
| Dimethylsulfoniopropionate demethylase | Granulosicoccus  Ruegeria | Kang et al., 2018  Wirth et al., 2020 |

References

Blasius, M., Hübscher, U., Sommer, S. (2008). *Deinococcus radiodurans*: What Belongs to the Survival Kit? Crit. Rev. Biochem. Mol. 43, 221-238.

Coates, C. J., & Wyman, M. (2017). A denitrifying community associated with a major, marine nitrogen fixer. Env microbiol, 19(12), 4978-4992.

Dang, H., Li, T., Chen, M., Huang, G. (2008). Cross-ocean distribution of rhodobacterales bacteria as primary surface colonizers in temperate coastal marine waters. Appl. Environ. Microbiol. 74, 52–60.

Huang, X.F., Liu, Y.J., Dong, J.D., Qu, L.Y., Zhang, Y.Y., Wang, F.Z., Tian, X.P., Zhang, S. (2014). *Mangrovibacterium diazotrophicum* gen. nov., sp. nov., a nitrogen-fixing bacterium isolated from a mangrove sediment, and proposal of Prolixibacteraceae fam. nov. Int. J. Syst. Evol. Microbiol. 64, 875–881.

Ivanova, E.G., Doronina, N.V., & Trotsenko, Y.A. (2001). Aerobic methylobacteria are capable of synthesizing auxins. Microbiology, *70*(4), 392-397.

Liu, Z., Frigaard, N.U., Vogl, K., Iino, T., Ohkuma, M., Overmann, J., & Bryant, D.A. (2012). Complete genome of Ignavibacterium album, a metabolically versatile, flagellated, facultative anaerobe from the phylum Chlorobi. Front Microbiol, 3, 185.

Kang, I., Lim, Y., Cho, J.C. (2018). Complete genome sequence of Granulosicoccus antarcticus type strain IMCC3135T, a marine gammaproteobacterium with a putative dimethylsulfoniopropionate demethylase gene. Mar. Genomics 37, 176–181.

Kumar, P.A., Srinivas, T.N. R., Thiel, V., Tank, M., Sasikala, C., Ramana, C. V., & Imhoff, J.F. (2009). Thiohalocapsa marina sp. nov., from an Indian marine aquaculture pond. International journal of systematic and evolutionary microbiology, 59(9), 2333-2338.

McClung, C.R., Patriquin, D.G., Davis, R.E. (1983). Campylobacter nitrofigilis sp. nov., a nitrogen-fixing bacterium associated with roots of Spartina alterniflora Loisel. Int. J. Syst. Bacteriol. 33, 605-612.

Mesa, J., Mateos-Naranjo, E., Caviedes, M.A., Redondo-Go´ mez, S., Pajuelo, E. & Rodrı´guez-Llorente, I.D., (2015). Endophytic cultivable bacteria of the metal bioaccumulator *Spartina maritima* improve plant growth but not metal uptake in polluted marshes soils. Front Microbiol 6, 1450.

Minamisawa, K., Nishioka, K., Miyaki, T., Ye, B., Miyamoto, T., You, M., & Sato, T. (2004). Anaerobic nitrogen-fixing consortia consisting of clostridia isolated from gramineous plants. Appl and env microbiol, 70(5), 3096-3102

Miroshnichenko, M.L., Kostrikina, N.A., Chernyh, N.A., Pimenov, N.V., Tourova, T.P., Antipov, A.N., Spring, S., Stackebrandt, E., Bonch-Osmolovskaya, E.A. (2003). *Caldithrix abyssi* gen. nov., sp. nov., a nitrate-reducing, thermophilic, anaerobic bacterium isolated from a Mid-Atlantic Ridge hydrothermal vent, represents a novel bacterial lineage. Int. J. Syst. Evol. Microbiol. 53, 323–329.

Nakagawa, T., Tsuchiya, Y., Ueda, S., Fukui, M., & Takahashi, R. (2018). Eelgrass sediment microbiome as a nitrous oxide sink in brackish lake Akkeshi, Japan. Microbes and environments, ME18103.

Prosser, J.I. [NITROGEN IN SOILS: Nitrification](https://www.sciencedirect.com/science/article/pii/B0123485304005129). In [Encyclopedia of Soils in the Environment](https://www.sciencedirect.com/referencework/9780123485304/encyclopedia-of-soils-in-the-environment), (2005).

Roalkvam, I., Drønen, K., Stokke, R., Daae, F. L., Dahle, H., & Steen, I. H. (2015). Physiological and genomic characterization of Arcobacter anaerophilus IR-1 reveals new metabolic features in Epsilonproteobacteria. Front Microbiol, *6*, 987.

Rua C.P.J., Thompson F. (2014) The Family *Cohaesibacteraceae*: The Genera *Cohaesibacter* and *Breoghania*. In: Rosenberg E., DeLong E.F., Lory S., Stackebrandt E., Thompson F. (eds) The Prokaryotes. Springer, Berlin, Heidelberg.

Sakami, T., Sakamoto, S., Takagi, S., Inaba, N., Imai, I. (2017). Distribution of three algicidal *Alteromonas* sp. strains in seagrass beds and surrounding areas in the Seto Inland Sea, Japan. Fish. Sci. 83, 113–121.

Straub, K.L., Rainey, F.A., & Widdel, F. (1999). Rhodovulum iodosum sp. nov. and Rhodovulum robiginosum sp. nov., two new marine phototrophic ferrous-iron-oxidizing purple bacteria. International Journal of Systematic and Evolutionary Microbiology, 49(2), 729-735.

Wirth, J.S., Wang, T., Huang, Q., White, R.H., Whitman, W.B. (2020). Dimethylsulfoniopropionate sulfur and methyl carbon assimilation in Ruegeria Species. mBio 11, e00329-20, /mbio/11/2/mBio.00329-20.atom.

Wu, Y.H., Xu, L., Meng, F.X., Zhang, D.S., Wang, C.S., Oren, A., Xu, X.W. (2014). *Altererythrobacter atlanticus* sp. nov., isolated from deep-sea sediment. Int. J. Syst. Evol. Microbiol. 64, 116–121

Zhang, W., Zhou, X., Yuan, Y., Liu, B., Epstein, S. S., & He, S. (2019). Complete Genome Sequence of Saccharospirillum mangrovi HK-33 T Sheds Light on the Ecological Role of a Bacterium in Mangrove Sediment Environment. Current microbiol, *76*(2), 231-236.

FAPROTAX

# Report for collapsed table: H_ovalis_functional_table.tsv

# Collapsed table generated on: 2021.03.10 12:43:44

# Original table: H_ovalis_FAPROTAX3.tsv

# Details:

# Original classical table contained 710 rows and 47 columns

# After filtering rows & columns based on indices, obtained a table containing 710 rows & 45 columns

# After (potentially) filtering out records based on name, obtained a table comprising 710 records & 45 data entries per record

# Used command:

# ./collapse_table.py -i H_ovalis_FAPROTAX3.tsv -o H_ovalis_functional_table.tsv -g FAPROTAX.txt -d taxonomy --omit_columns 0 -r H_ovalis_report.txt -v --force

# Summary of group assignments:

# methanotrophy: 2 records

# acetoclastic_methanogenesis: 0 records

# methanogenesis_by_disproportionation_of_methyl_groups: 0 records

# methanogenesis_using_formate: 0 records

# methanogenesis_by_CO2_reduction_with_H2: 0 records

# methanogenesis_by_reduction_of_methyl_compounds_with_H2: 0 records

# hydrogenotrophic_methanogenesis: 0 records

# methanogenesis: 0 records

# methanol_oxidation: 9 records

# methylotrophy: 11 records

# aerobic_ammonia_oxidation: 1 records

# aerobic_nitrite_oxidation: 1 records

# nitrification: 2 records

# sulfate_respiration: 100 records

# sulfur_respiration: 0 records

# dark_sulfite_oxidation: 2 records

# sulfite_respiration: 9 records

# thiosulfate_respiration: 1 records

# respiration_of_sulfur_compounds: 100 records

# arsenate_detoxification: 0 records

# arsenate_respiration: 0 records

# dissimilatory_arsenate_reduction: 0 records

# arsenite_oxidation_detoxification: 0 records

# arsenite_oxidation_energy_yielding: 0 records

# dissimilatory_arsenite_oxidation: 0 records

# anammox: 0 records

# nitrate_denitrification: 0 records

# nitrite_denitrification: 0 records

# nitrous_oxide_denitrification: 0 records

# denitrification: 0 records

# chitinolysis: 1 records

# knallgas_bacteria: 0 records

# dark_hydrogen_oxidation: 2 records

# nitrogen_fixation: 1 records

# nitrate_ammonification: 0 records

# nitrite_ammonification: 0 records

# nitrite_respiration: 0 records

# cellulolysis: 9 records

# xylanolysis: 1 records

# dark_sulfide_oxidation: 2 records

# dark_sulfur_oxidation: 3 records

# dark_thiosulfate_oxidation: 2 records

# dark_oxidation_of_sulfur_compounds: 9 records

# manganese_oxidation: 0 records

# manganese_respiration: 0 records

# ligninolysis: 0 records

# fermentation: 35 records

# aerobic_chemoheterotrophy: 111 records

# invertebrate_parasites: 0 records

# human_pathogens_septicemia: 0 records

# human_pathogens_pneumonia: 0 records

# human_pathogens_nosocomia: 0 records

# human_pathogens_meningitis: 0 records

# human_pathogens_gastroenteritis: 0 records

# human_pathogens_diarrhea: 0 records

# human_pathogens_all: 0 records

# fish_parasites: 0 records

# human_gut: 0 records

# human_associated: 0 records

# mammal_gut: 0 records

# animal_parasites_or_symbionts: 0 records

# plant_pathogen: 0 records

# oil_bioremediation: 0 records

# aromatic_hydrocarbon_degradation: 3 records

# aromatic_compound_degradation: 6 records

# aliphatic_non_methane_hydrocarbon_degradation: 0 records

# hydrocarbon_degradation: 5 records

# dark_iron_oxidation: 0 records

# iron_respiration: 0 records

# nitrate_respiration: 0 records

# nitrate_reduction: 2 records

# nitrogen_respiration: 0 records

# fumarate_respiration: 0 records

# intracellular_parasites: 2 records

# chlorate_reducers: 0 records

# predatory_or_exoparasitic: 2 records

# chloroplasts: 0 records

# nonphotosynthetic_cyanobacteria: 0 records

# photosynthetic_cyanobacteria: 0 records

# anoxygenic_photoautotrophy_H2_oxidizing: 0 records

# anoxygenic_photoautotrophy_S_oxidizing: 8 records

# anoxygenic_photoautotrophy_Fe_oxidizing: 0 records

# anoxygenic_photoautotrophy: 10 records

# oxygenic_photoautotrophy: 0 records

# photoautotrophy: 10 records

# aerobic_anoxygenic_phototrophy: 0 records

# photoheterotrophy: 3 records

# phototrophy: 11 records

# plastic_degradation: 0 records

# ureolysis: 2 records

# reductive_acetogenesis: 0 records

# chemoheterotrophy: 148 records

#

# Loaded 92 groups comprising 8312 members (5044 unique members)

# Established 626 assignments of records to groups

# 255 out of 710 records (35.9155 %) were assigned to at least one group

# 455 out of 710 records (64.0845 %) could not be assigned to any group (leftovers)

# 35 groups were represented (i.e. associated with at least one record)

# Detailed group assignments are listed below

# methanotrophy (2 records):

Proteobacteria; Alphaproteobacteria; Rhizobiales; Methylocystaceae; Methylocystis

Proteobacteria; Alphaproteobacteria; Rhizobiales; Methylocystaceae; Terasakiella

# acetoclastic_methanogenesis (0 records):

# methanogenesis_by_disproportionation_of_methyl_groups (0 records):

# methanogenesis_using_formate (0 records):

# methanogenesis_by_CO2_reduction_with_H2 (0 records):

# methanogenesis_by_reduction_of_methyl_compounds_with_H2 (0 records):

# hydrogenotrophic_methanogenesis (0 records):

# methanogenesis (0 records):

# methanol_oxidation (9 records):

Proteobacteria; Betaproteobacteria; Methylophilales; Methylophilaceae; unclassified

Proteobacteria; Betaproteobacteria; Methylophilales; Methylophilaceae; unclassified

Proteobacteria; Betaproteobacteria; Methylophilales; Methylophilaceae; unclassified

Proteobacteria; Betaproteobacteria; Methylophilales; Methylophilaceae; unclassified

Proteobacteria; Betaproteobacteria; Methylophilales; Methylophilaceae; unclassified

Proteobacteria; Gammaproteobacteria; Thiotrichales; Piscirickettsiaceae; Methylophaga

Proteobacteria; Gammaproteobacteria; Thiotrichales; Piscirickettsiaceae; Methylophaga

Proteobacteria; Gammaproteobacteria; Thiotrichales; Piscirickettsiaceae; Methylophaga

Proteobacteria; Gammaproteobacteria; Thiotrichales; Piscirickettsiaceae; Methylophaga

# methylotrophy (11 records):

Proteobacteria; Alphaproteobacteria; Rhizobiales; Methylocystaceae; Methylocystis

Proteobacteria; Alphaproteobacteria; Rhizobiales; Methylocystaceae; Terasakiella

Proteobacteria; Betaproteobacteria; Methylophilales; Methylophilaceae; unclassified

Proteobacteria; Betaproteobacteria; Methylophilales; Methylophilaceae; unclassified

Proteobacteria; Betaproteobacteria; Methylophilales; Methylophilaceae; unclassified

Proteobacteria; Betaproteobacteria; Methylophilales; Methylophilaceae; unclassified

Proteobacteria; Betaproteobacteria; Methylophilales; Methylophilaceae; unclassified

Proteobacteria; Gammaproteobacteria; Thiotrichales; Piscirickettsiaceae; Methylophaga

Proteobacteria; Gammaproteobacteria; Thiotrichales; Piscirickettsiaceae; Methylophaga

Proteobacteria; Gammaproteobacteria; Thiotrichales; Piscirickettsiaceae; Methylophaga

Proteobacteria; Gammaproteobacteria; Thiotrichales; Piscirickettsiaceae; Methylophaga

# aerobic_ammonia_oxidation (1 records):

Proteobacteria; Gammaproteobacteria; Chromatiales; Chromatiaceae; Nitrosococcus

# aerobic_nitrite_oxidation (1 records):

Nitrospirae; Nitrospira; Nitrospirales; Nitrospiraceae; unclassified

# nitrification (2 records):

Nitrospirae; Nitrospira; Nitrospirales; Nitrospiraceae; unclassified

Proteobacteria; Gammaproteobacteria; Chromatiales; Chromatiaceae; Nitrosococcus

# sulfate_respiration (100 records):

Proteobacteria; Deltaproteobacteria; Desulfobacterales; Desulfobacteraceae; Desulfobacter

Proteobacteria; Deltaproteobacteria; Desulfobacterales; Desulfobacteraceae; Desulfobacterium

Proteobacteria; Deltaproteobacteria; Desulfobacterales; Desulfobacteraceae; Desulfosarcina

Proteobacteria; Deltaproteobacteria; Desulfobacterales; Desulfobacteraceae; Desulfosarcina

Proteobacteria; Deltaproteobacteria; Desulfobacterales; Desulfobacteraceae; Desulfosarcina

Proteobacteria; Deltaproteobacteria; Desulfobacterales; Desulfobacteraceae; Desulfosarcina

Proteobacteria; Deltaproteobacteria; Desulfobacterales; Desulfobacteraceae; Desulfosarcina

Proteobacteria; Deltaproteobacteria; Desulfobacterales; Desulfobacteraceae; Desulfosarcina

Proteobacteria; Deltaproteobacteria; Desulfobacterales; Desulfobacteraceae; Desulfosarcina

Proteobacteria; Deltaproteobacteria; Desulfobacterales; Desulfobacteraceae; Desulfosarcina

Proteobacteria; Deltaproteobacteria; Desulfobacterales; Desulfobacteraceae; unclassified

Proteobacteria; Deltaproteobacteria; Desulfobacterales; Desulfobacteraceae; unclassified

Proteobacteria; Deltaproteobacteria; Desulfobacterales; Desulfobacteraceae; unclassified

Proteobacteria; Deltaproteobacteria; Desulfobacterales; Desulfobacteraceae; unclassified

Proteobacteria; Deltaproteobacteria; Desulfobacterales; Desulfobacteraceae; unclassified

Proteobacteria; Deltaproteobacteria; Desulfobacterales; Desulfobacteraceae; unclassified

Proteobacteria; Deltaproteobacteria; Desulfobacterales; Desulfobacteraceae; unclassified

Proteobacteria; Deltaproteobacteria; Desulfobacterales; Desulfobacteraceae; unclassified

Proteobacteria; Deltaproteobacteria; Desulfobacterales; Desulfobacteraceae; unclassified

Proteobacteria; Deltaproteobacteria; Desulfobacterales; Desulfobacteraceae; unclassified

Proteobacteria; Deltaproteobacteria; Desulfobacterales; Desulfobacteraceae; unclassified

Proteobacteria; Deltaproteobacteria; Desulfobacterales; Desulfobacteraceae; unclassified

Proteobacteria; Deltaproteobacteria; Desulfobacterales; Desulfobacteraceae; unclassified

Proteobacteria; Deltaproteobacteria; Desulfobacterales; Desulfobacteraceae; unclassified

Proteobacteria; Deltaproteobacteria; Desulfobacterales; Desulfobacteraceae; unclassified

Proteobacteria; Deltaproteobacteria; Desulfobacterales; Desulfobacteraceae; unclassified

Proteobacteria; Deltaproteobacteria; Desulfobacterales; Desulfobacteraceae; unclassified

Proteobacteria; Deltaproteobacteria; Desulfobacterales; Desulfobacteraceae; unclassified

Proteobacteria; Deltaproteobacteria; Desulfobacterales; Desulfobacteraceae; unclassified

Proteobacteria; Deltaproteobacteria; Desulfobacterales; Desulfobacteraceae; unclassified

Proteobacteria; Deltaproteobacteria; Desulfobacterales; Desulfobacteraceae; unclassified

Proteobacteria; Deltaproteobacteria; Desulfobacterales; Desulfobacteraceae; unclassified

Proteobacteria; Deltaproteobacteria; Desulfobacterales; Desulfobacteraceae; unclassified

Proteobacteria; Deltaproteobacteria; Desulfobacterales; Desulfobacteraceae; unclassified

Proteobacteria; Deltaproteobacteria; Desulfobacterales; Desulfobacteraceae; unclassified

Proteobacteria; Deltaproteobacteria; Desulfobacterales; Desulfobacteraceae; unclassified

Proteobacteria; Deltaproteobacteria; Desulfobacterales; Desulfobulbaceae; Desulfobulbus

Proteobacteria; Deltaproteobacteria; Desulfobacterales; Desulfobulbaceae; Desulfobulbus

Proteobacteria; Deltaproteobacteria; Desulfobacterales; Desulfobulbaceae; Desulfobulbus

Proteobacteria; Deltaproteobacteria; Desulfobacterales; Desulfobulbaceae; Desulfobulbus

Proteobacteria; Deltaproteobacteria; Desulfobacterales; Desulfobulbaceae; Desulfobulbus

Proteobacteria; Deltaproteobacteria; Desulfobacterales; Desulfobulbaceae; Desulfobulbus

Proteobacteria; Deltaproteobacteria; Desulfobacterales; Desulfobulbaceae; Desulfobulbus

Proteobacteria; Deltaproteobacteria; Desulfobacterales; Desulfobulbaceae; Desulfobulbus

Proteobacteria; Deltaproteobacteria; Desulfobacterales; Desulfobulbaceae; Desulfofustis

Proteobacteria; Deltaproteobacteria; Desulfobacterales; Desulfobulbaceae; Desulfopila

Proteobacteria; Deltaproteobacteria; Desulfobacterales; Desulfobulbaceae; Desulfopila

Proteobacteria; Deltaproteobacteria; Desulfobacterales; Desulfobulbaceae; Desulfopila

Proteobacteria; Deltaproteobacteria; Desulfobacterales; Desulfobulbaceae; Desulfopila

Proteobacteria; Deltaproteobacteria; Desulfobacterales; Desulfobulbaceae; Desulfopila

Proteobacteria; Deltaproteobacteria; Desulfobacterales; Desulfobulbaceae; Desulfopila

Proteobacteria; Deltaproteobacteria; Desulfobacterales; Desulfobulbaceae; Desulfopila

Proteobacteria; Deltaproteobacteria; Desulfobacterales; Desulfobulbaceae; Desulfopila

Proteobacteria; Deltaproteobacteria; Desulfobacterales; Desulfobulbaceae; Desulfopila

Proteobacteria; Deltaproteobacteria; Desulfobacterales; Desulfobulbaceae; Desulforhopalus

Proteobacteria; Deltaproteobacteria; Desulfobacterales; Desulfobulbaceae; Desulforhopalus

Proteobacteria; Deltaproteobacteria; Desulfobacterales; Desulfobulbaceae; Desulfotalea

Proteobacteria; Deltaproteobacteria; Desulfobacterales; Desulfobulbaceae; Desulfotalea

Proteobacteria; Deltaproteobacteria; Desulfobacterales; Desulfobulbaceae; Desulfotalea

Proteobacteria; Deltaproteobacteria; Desulfobacterales; Desulfobulbaceae; Desulfotalea

Proteobacteria; Deltaproteobacteria; Desulfobacterales; Desulfobulbaceae; Desulfotalea

Proteobacteria; Deltaproteobacteria; Desulfobacterales; Desulfobulbaceae; Desulfotalea

Proteobacteria; Deltaproteobacteria; Desulfobacterales; Desulfobulbaceae; unclassified

Proteobacteria; Deltaproteobacteria; Desulfobacterales; Desulfobulbaceae; unclassified

Proteobacteria; Deltaproteobacteria; Desulfobacterales; Desulfobulbaceae; unclassified

Proteobacteria; Deltaproteobacteria; Desulfobacterales; Desulfobulbaceae; unclassified

Proteobacteria; Deltaproteobacteria; Desulfobacterales; Desulfobulbaceae; unclassified

Proteobacteria; Deltaproteobacteria; Desulfobacterales; Desulfobulbaceae; unclassified

Proteobacteria; Deltaproteobacteria; Desulfobacterales; Desulfobulbaceae; unclassified

Proteobacteria; Deltaproteobacteria; Desulfobacterales; Desulfobulbaceae; unclassified

Proteobacteria; Deltaproteobacteria; Desulfobacterales; Desulfobulbaceae; unclassified

Proteobacteria; Deltaproteobacteria; Desulfobacterales; Desulfobulbaceae; unclassified

Proteobacteria; Deltaproteobacteria; Desulfobacterales; Desulfobulbaceae; unclassified

Proteobacteria; Deltaproteobacteria; Desulfobacterales; Desulfobulbaceae; unclassified

Proteobacteria; Deltaproteobacteria; Desulfobacterales; Desulfobulbaceae; unclassified

Proteobacteria; Deltaproteobacteria; Desulfobacterales; Desulfobulbaceae; unclassified

Proteobacteria; Deltaproteobacteria; Desulfobacterales; Desulfobulbaceae; unclassified

Proteobacteria; Deltaproteobacteria; Desulfobacterales; Desulfobulbaceae; unclassified

Proteobacteria; Deltaproteobacteria; Desulfobacterales; Desulfobulbaceae; unclassified

Proteobacteria; Deltaproteobacteria; Desulfobacterales; Desulfobulbaceae; unclassified

Proteobacteria; Deltaproteobacteria; Desulfobacterales; Desulfobulbaceae; unclassified

Proteobacteria; Deltaproteobacteria; Desulfobacterales; Desulfobulbaceae; unclassified

Proteobacteria; Deltaproteobacteria; Desulfobacterales; Desulfobulbaceae; unclassified

Proteobacteria; Deltaproteobacteria; Desulfobacterales; Desulfobulbaceae; unclassified

Proteobacteria; Deltaproteobacteria; Desulfobacterales; Desulfobulbaceae; unclassified

Proteobacteria; Deltaproteobacteria; Desulfobacterales; Desulfobulbaceae; unclassified

Proteobacteria; Deltaproteobacteria; Desulfobacterales; Desulfobulbaceae; unclassified

Proteobacteria; Deltaproteobacteria; Desulfobacterales; Desulfobulbaceae; unclassified

Proteobacteria; Deltaproteobacteria; Desulfobacterales; Desulfobulbaceae; unclassified

Proteobacteria; Deltaproteobacteria; Desulfobacterales; Desulfobulbaceae; unclassified

Proteobacteria; Deltaproteobacteria; Desulfobacterales; Desulfobulbaceae; unclassified

Proteobacteria; Deltaproteobacteria; Desulfobacterales; Desulfobulbaceae; unclassified

Proteobacteria; Deltaproteobacteria; Desulfobacterales; Desulfobulbaceae; unclassified

Proteobacteria; Deltaproteobacteria; Desulfobacterales; Desulfobulbaceae; unclassified

Proteobacteria; Deltaproteobacteria; Desulfobacterales; Desulfobulbaceae; unclassified

Proteobacteria; Deltaproteobacteria; Desulfobacterales; Desulfobulbaceae; unclassified

Proteobacteria; Deltaproteobacteria; Desulfovibrionales; Desulfohalobiaceae; unclassified

Proteobacteria; Deltaproteobacteria; Desulfovibrionales; Desulfovibrionaceae; Desulfocurvus

Proteobacteria; Deltaproteobacteria; Desulfovibrionales; Desulfovibrionaceae; Desulfovibrio

Proteobacteria; Deltaproteobacteria; Desulfovibrionales; Desulfovibrionaceae; Desulfovibrio

# sulfur_respiration (0 records):

# dark_sulfite_oxidation (2 records):

Proteobacteria; Alphaproteobacteria; Rhodobacterales; Rhodobacteraceae; Sulfitobacter

Proteobacteria; Alphaproteobacteria; Rhodobacterales; Rhodobacteraceae; Sulfitobacter

# sulfite_respiration (9 records):

Proteobacteria; Deltaproteobacteria; Desulfobacterales; Desulfobacteraceae; Desulfobacter

Proteobacteria; Deltaproteobacteria; Desulfobacterales; Desulfobulbaceae; Desulfobulbus

Proteobacteria; Deltaproteobacteria; Desulfobacterales; Desulfobulbaceae; Desulfobulbus

Proteobacteria; Deltaproteobacteria; Desulfobacterales; Desulfobulbaceae; Desulfobulbus

Proteobacteria; Deltaproteobacteria; Desulfobacterales; Desulfobulbaceae; Desulfobulbus

Proteobacteria; Deltaproteobacteria; Desulfobacterales; Desulfobulbaceae; Desulfobulbus

Proteobacteria; Deltaproteobacteria; Desulfobacterales; Desulfobulbaceae; Desulfobulbus

Proteobacteria; Deltaproteobacteria; Desulfobacterales; Desulfobulbaceae; Desulfobulbus

Proteobacteria; Deltaproteobacteria; Desulfobacterales; Desulfobulbaceae; Desulfobulbus

# thiosulfate_respiration (1 records):

Proteobacteria; Deltaproteobacteria; Desulfobacterales; Desulfobacteraceae; Desulfobacter

# respiration_of_sulfur_compounds (100 records):

Proteobacteria; Deltaproteobacteria; Desulfobacterales; Desulfobacteraceae; Desulfobacter

Proteobacteria; Deltaproteobacteria; Desulfobacterales; Desulfobacteraceae; Desulfobacterium

Proteobacteria; Deltaproteobacteria; Desulfobacterales; Desulfobacteraceae; Desulfosarcina

Proteobacteria; Deltaproteobacteria; Desulfobacterales; Desulfobacteraceae; Desulfosarcina

Proteobacteria; Deltaproteobacteria; Desulfobacterales; Desulfobacteraceae; Desulfosarcina

Proteobacteria; Deltaproteobacteria; Desulfobacterales; Desulfobacteraceae; Desulfosarcina

Proteobacteria; Deltaproteobacteria; Desulfobacterales; Desulfobacteraceae; Desulfosarcina

Proteobacteria; Deltaproteobacteria; Desulfobacterales; Desulfobacteraceae; Desulfosarcina

Proteobacteria; Deltaproteobacteria; Desulfobacterales; Desulfobacteraceae; Desulfosarcina

Proteobacteria; Deltaproteobacteria; Desulfobacterales; Desulfobacteraceae; Desulfosarcina

Proteobacteria; Deltaproteobacteria; Desulfobacterales; Desulfobacteraceae; unclassified

Proteobacteria; Deltaproteobacteria; Desulfobacterales; Desulfobacteraceae; unclassified

Proteobacteria; Deltaproteobacteria; Desulfobacterales; Desulfobacteraceae; unclassified

Proteobacteria; Deltaproteobacteria; Desulfobacterales; Desulfobacteraceae; unclassified

Proteobacteria; Deltaproteobacteria; Desulfobacterales; Desulfobacteraceae; unclassified

Proteobacteria; Deltaproteobacteria; Desulfobacterales; Desulfobacteraceae; unclassified

Proteobacteria; Deltaproteobacteria; Desulfobacterales; Desulfobacteraceae; unclassified

Proteobacteria; Deltaproteobacteria; Desulfobacterales; Desulfobacteraceae; unclassified

Proteobacteria; Deltaproteobacteria; Desulfobacterales; Desulfobacteraceae; unclassified

Proteobacteria; Deltaproteobacteria; Desulfobacterales; Desulfobacteraceae; unclassified

Proteobacteria; Deltaproteobacteria; Desulfobacterales; Desulfobacteraceae; unclassified

Proteobacteria; Deltaproteobacteria; Desulfobacterales; Desulfobacteraceae; unclassified

Proteobacteria; Deltaproteobacteria; Desulfobacterales; Desulfobacteraceae; unclassified

Proteobacteria; Deltaproteobacteria; Desulfobacterales; Desulfobacteraceae; unclassified

Proteobacteria; Deltaproteobacteria; Desulfobacterales; Desulfobacteraceae; unclassified

Proteobacteria; Deltaproteobacteria; Desulfobacterales; Desulfobacteraceae; unclassified

Proteobacteria; Deltaproteobacteria; Desulfobacterales; Desulfobacteraceae; unclassified

Proteobacteria; Deltaproteobacteria; Desulfobacterales; Desulfobacteraceae; unclassified

Proteobacteria; Deltaproteobacteria; Desulfobacterales; Desulfobacteraceae; unclassified

Proteobacteria; Deltaproteobacteria; Desulfobacterales; Desulfobacteraceae; unclassified

Proteobacteria; Deltaproteobacteria; Desulfobacterales; Desulfobacteraceae; unclassified

Proteobacteria; Deltaproteobacteria; Desulfobacterales; Desulfobacteraceae; unclassified

Proteobacteria; Deltaproteobacteria; Desulfobacterales; Desulfobacteraceae; unclassified

Proteobacteria; Deltaproteobacteria; Desulfobacterales; Desulfobacteraceae; unclassified

Proteobacteria; Deltaproteobacteria; Desulfobacterales; Desulfobacteraceae; unclassified

Proteobacteria; Deltaproteobacteria; Desulfobacterales; Desulfobacteraceae; unclassified

Proteobacteria; Deltaproteobacteria; Desulfobacterales; Desulfobulbaceae; Desulfobulbus

Proteobacteria; Deltaproteobacteria; Desulfobacterales; Desulfobulbaceae; Desulfobulbus

Proteobacteria; Deltaproteobacteria; Desulfobacterales; Desulfobulbaceae; Desulfobulbus

Proteobacteria; Deltaproteobacteria; Desulfobacterales; Desulfobulbaceae; Desulfobulbus

Proteobacteria; Deltaproteobacteria; Desulfobacterales; Desulfobulbaceae; Desulfobulbus

Proteobacteria; Deltaproteobacteria; Desulfobacterales; Desulfobulbaceae; Desulfobulbus

Proteobacteria; Deltaproteobacteria; Desulfobacterales; Desulfobulbaceae; Desulfobulbus

Proteobacteria; Deltaproteobacteria; Desulfobacterales; Desulfobulbaceae; Desulfobulbus

Proteobacteria; Deltaproteobacteria; Desulfobacterales; Desulfobulbaceae; Desulfofustis

Proteobacteria; Deltaproteobacteria; Desulfobacterales; Desulfobulbaceae; Desulfopila

Proteobacteria; Deltaproteobacteria; Desulfobacterales; Desulfobulbaceae; Desulfopila

Proteobacteria; Deltaproteobacteria; Desulfobacterales; Desulfobulbaceae; Desulfopila

Proteobacteria; Deltaproteobacteria; Desulfobacterales; Desulfobulbaceae; Desulfopila

Proteobacteria; Deltaproteobacteria; Desulfobacterales; Desulfobulbaceae; Desulfopila

Proteobacteria; Deltaproteobacteria; Desulfobacterales; Desulfobulbaceae; Desulfopila

Proteobacteria; Deltaproteobacteria; Desulfobacterales; Desulfobulbaceae; Desulfopila

Proteobacteria; Deltaproteobacteria; Desulfobacterales; Desulfobulbaceae; Desulfopila

Proteobacteria; Deltaproteobacteria; Desulfobacterales; Desulfobulbaceae; Desulfopila

Proteobacteria; Deltaproteobacteria; Desulfobacterales; Desulfobulbaceae; Desulforhopalus

Proteobacteria; Deltaproteobacteria; Desulfobacterales; Desulfobulbaceae; Desulforhopalus

Proteobacteria; Deltaproteobacteria; Desulfobacterales; Desulfobulbaceae; Desulfotalea

Proteobacteria; Deltaproteobacteria; Desulfobacterales; Desulfobulbaceae; Desulfotalea

Proteobacteria; Deltaproteobacteria; Desulfobacterales; Desulfobulbaceae; Desulfotalea

Proteobacteria; Deltaproteobacteria; Desulfobacterales; Desulfobulbaceae; Desulfotalea

Proteobacteria; Deltaproteobacteria; Desulfobacterales; Desulfobulbaceae; Desulfotalea

Proteobacteria; Deltaproteobacteria; Desulfobacterales; Desulfobulbaceae; Desulfotalea

Proteobacteria; Deltaproteobacteria; Desulfobacterales; Desulfobulbaceae; unclassified

Proteobacteria; Deltaproteobacteria; Desulfobacterales; Desulfobulbaceae; unclassified

Proteobacteria; Deltaproteobacteria; Desulfobacterales; Desulfobulbaceae; unclassified

Proteobacteria; Deltaproteobacteria; Desulfobacterales; Desulfobulbaceae; unclassified

Proteobacteria; Deltaproteobacteria; Desulfobacterales; Desulfobulbaceae; unclassified

Proteobacteria; Deltaproteobacteria; Desulfobacterales; Desulfobulbaceae; unclassified

Proteobacteria; Deltaproteobacteria; Desulfobacterales; Desulfobulbaceae; unclassified

Proteobacteria; Deltaproteobacteria; Desulfobacterales; Desulfobulbaceae; unclassified

Proteobacteria; Deltaproteobacteria; Desulfobacterales; Desulfobulbaceae; unclassified

Proteobacteria; Deltaproteobacteria; Desulfobacterales; Desulfobulbaceae; unclassified

Proteobacteria; Deltaproteobacteria; Desulfobacterales; Desulfobulbaceae; unclassified

Proteobacteria; Deltaproteobacteria; Desulfobacterales; Desulfobulbaceae; unclassified

Proteobacteria; Deltaproteobacteria; Desulfobacterales; Desulfobulbaceae; unclassified

Proteobacteria; Deltaproteobacteria; Desulfobacterales; Desulfobulbaceae; unclassified

Proteobacteria; Deltaproteobacteria; Desulfobacterales; Desulfobulbaceae; unclassified

Proteobacteria; Deltaproteobacteria; Desulfobacterales; Desulfobulbaceae; unclassified

Proteobacteria; Deltaproteobacteria; Desulfobacterales; Desulfobulbaceae; unclassified

Proteobacteria; Deltaproteobacteria; Desulfobacterales; Desulfobulbaceae; unclassified

Proteobacteria; Deltaproteobacteria; Desulfobacterales; Desulfobulbaceae; unclassified

Proteobacteria; Deltaproteobacteria; Desulfobacterales; Desulfobulbaceae; unclassified

Proteobacteria; Deltaproteobacteria; Desulfobacterales; Desulfobulbaceae; unclassified

Proteobacteria; Deltaproteobacteria; Desulfobacterales; Desulfobulbaceae; unclassified

Proteobacteria; Deltaproteobacteria; Desulfobacterales; Desulfobulbaceae; unclassified

Proteobacteria; Deltaproteobacteria; Desulfobacterales; Desulfobulbaceae; unclassified

Proteobacteria; Deltaproteobacteria; Desulfobacterales; Desulfobulbaceae; unclassified

Proteobacteria; Deltaproteobacteria; Desulfobacterales; Desulfobulbaceae; unclassified

Proteobacteria; Deltaproteobacteria; Desulfobacterales; Desulfobulbaceae; unclassified

Proteobacteria; Deltaproteobacteria; Desulfobacterales; Desulfobulbaceae; unclassified

Proteobacteria; Deltaproteobacteria; Desulfobacterales; Desulfobulbaceae; unclassified

Proteobacteria; Deltaproteobacteria; Desulfobacterales; Desulfobulbaceae; unclassified

Proteobacteria; Deltaproteobacteria; Desulfobacterales; Desulfobulbaceae; unclassified

Proteobacteria; Deltaproteobacteria; Desulfobacterales; Desulfobulbaceae; unclassified

Proteobacteria; Deltaproteobacteria; Desulfobacterales; Desulfobulbaceae; unclassified

Proteobacteria; Deltaproteobacteria; Desulfobacterales; Desulfobulbaceae; unclassified

Proteobacteria; Deltaproteobacteria; Desulfovibrionales; Desulfohalobiaceae; unclassified

Proteobacteria; Deltaproteobacteria; Desulfovibrionales; Desulfovibrionaceae; Desulfocurvus

Proteobacteria; Deltaproteobacteria; Desulfovibrionales; Desulfovibrionaceae; Desulfovibrio

Proteobacteria; Deltaproteobacteria; Desulfovibrionales; Desulfovibrionaceae; Desulfovibrio

# arsenate_detoxification (0 records):

# arsenate_respiration (0 records):

# dissimilatory_arsenate_reduction (0 records):

# arsenite_oxidation_detoxification (0 records):

# arsenite_oxidation_energy_yielding (0 records):

# dissimilatory_arsenite_oxidation (0 records):

# anammox (0 records):

# nitrate_denitrification (0 records):

# nitrite_denitrification (0 records):

# nitrous_oxide_denitrification (0 records):

# denitrification (0 records):

# chitinolysis (1 records):

Proteobacteria; Gammaproteobacteria; Alteromonadales; Alteromonadaceae; Microbulbifer

# knallgas_bacteria (0 records):

# dark_hydrogen_oxidation (2 records):

Proteobacteria; Deltaproteobacteria; Desulfobacterales; Desulfobulbaceae; Desulforhopalus

Proteobacteria; Deltaproteobacteria; Desulfobacterales; Desulfobulbaceae; Desulforhopalus

# nitrogen_fixation (1 records):

Proteobacteria; Alphaproteobacteria; Rhizobiales; Methylocystaceae; Methylocystis

# nitrate_ammonification (0 records):

# nitrite_ammonification (0 records):

# nitrite_respiration (0 records):

# cellulolysis (9 records):

Bacteroidetes; Sphingobacteriia; Sphingobacteriales; Saprospiraceae; Lewinella

Bacteroidetes; Sphingobacteriia; Sphingobacteriales; Saprospiraceae; Lewinella

Bacteroidetes; Sphingobacteriia; Sphingobacteriales; Saprospiraceae; Lewinella

Bacteroidetes; Sphingobacteriia; Sphingobacteriales; Saprospiraceae; Lewinella

Bacteroidetes; Sphingobacteriia; Sphingobacteriales; Saprospiraceae; Lewinella

Bacteroidetes; Sphingobacteriia; Sphingobacteriales; Saprospiraceae; Lewinella

Bacteroidetes; Sphingobacteriia; Sphingobacteriales; Saprospiraceae; Lewinella

Fibrobacteres; Fibrobacteria; Fibrobacterales; Fibrobacteraceae; Fibrobacter

Proteobacteria; Gammaproteobacteria; Alteromonadales; Alteromonadaceae; Microbulbifer

# xylanolysis (1 records):

Proteobacteria; Gammaproteobacteria; Alteromonadales; Alteromonadaceae; Microbulbifer

# dark_sulfide_oxidation (2 records):

Proteobacteria; Gammaproteobacteria; Chromatiales; Ectothiorhodospiraceae; Thioalkalivibrio

Proteobacteria; Gammaproteobacteria; Thiotrichales; Thiotrichaceae; Thiothrix

# dark_sulfur_oxidation (3 records):

Proteobacteria; Alphaproteobacteria; Rhodobacterales; Rhodobacteraceae; Sulfitobacter

Proteobacteria; Alphaproteobacteria; Rhodobacterales; Rhodobacteraceae; Sulfitobacter

Proteobacteria; Gammaproteobacteria; Chromatiales; Ectothiorhodospiraceae; Thioalkalivibrio

# dark_thiosulfate_oxidation (2 records):

Proteobacteria; Alphaproteobacteria; Rhodobacterales; Rhodobacteraceae; Citreicella

Proteobacteria; Gammaproteobacteria; Chromatiales; Ectothiorhodospiraceae; Thioalkalivibrio

# dark_oxidation_of_sulfur_compounds (9 records):

Proteobacteria; Alphaproteobacteria; Rhodobacterales; Rhodobacteraceae; Citreicella

Proteobacteria; Alphaproteobacteria; Rhodobacterales; Rhodobacteraceae; Sulfitobacter

Proteobacteria; Alphaproteobacteria; Rhodobacterales; Rhodobacteraceae; Sulfitobacter

Proteobacteria; Epsilonproteobacteria; Campylobacterales; Helicobacteraceae; Sulfurimonas

Proteobacteria; Epsilonproteobacteria; Campylobacterales; Helicobacteraceae; Sulfurimonas

Proteobacteria; Epsilonproteobacteria; Campylobacterales; Helicobacteraceae; Sulfurimonas

Proteobacteria; Epsilonproteobacteria; Campylobacterales; Helicobacteraceae; Sulfurimonas

Proteobacteria; Gammaproteobacteria; Chromatiales; Ectothiorhodospiraceae; Thioalkalivibrio

Proteobacteria; Gammaproteobacteria; Thiotrichales; Thiotrichaceae; Thiothrix

# manganese_oxidation (0 records):

# manganese_respiration (0 records):

# ligninolysis (0 records):

# fermentation (35 records):

Actinobacteria; Actinobacteria; Actinomycetales; Propionibacteriaceae; Propionibacterium

Bacteroidetes; Flavobacteriia; Flavobacteriales; Flavobacteriaceae; Muricauda

Bacteroidetes; Flavobacteriia; Flavobacteriales; Flavobacteriaceae; Muricauda

Fibrobacteres; Fibrobacteria; Fibrobacterales; Fibrobacteraceae; Fibrobacter

Proteobacteria; Alphaproteobacteria; Rhodobacterales; Rhodobacteraceae; Rhodovulum

Proteobacteria; Alphaproteobacteria; Rhodobacterales; Rhodobacteraceae; Rhodovulum

Proteobacteria; Deltaproteobacteria; Desulfobacterales; Desulfobacteraceae; Desulfobacterium

Proteobacteria; Deltaproteobacteria; Desulfobacterales; Desulfobulbaceae; Desulfobulbus

Proteobacteria; Deltaproteobacteria; Desulfobacterales; Desulfobulbaceae; Desulfobulbus

Proteobacteria; Deltaproteobacteria; Desulfobacterales; Desulfobulbaceae; Desulfobulbus

Proteobacteria; Deltaproteobacteria; Desulfobacterales; Desulfobulbaceae; Desulfobulbus

Proteobacteria; Deltaproteobacteria; Desulfobacterales; Desulfobulbaceae; Desulfobulbus

Proteobacteria; Deltaproteobacteria; Desulfobacterales; Desulfobulbaceae; Desulfobulbus

Proteobacteria; Deltaproteobacteria; Desulfobacterales; Desulfobulbaceae; Desulfobulbus

Proteobacteria; Deltaproteobacteria; Desulfobacterales; Desulfobulbaceae; Desulfobulbus

Proteobacteria; Deltaproteobacteria; Desulfobacterales; Desulfobulbaceae; Desulforhopalus

Proteobacteria; Deltaproteobacteria; Desulfobacterales; Desulfobulbaceae; Desulforhopalus

Proteobacteria; Deltaproteobacteria; Desulfovibrionales; Desulfovibrionaceae; Desulfocurvus

Proteobacteria; Deltaproteobacteria; Desulfuromonadales; Desulfuromonadaceae; Malonomonas

Proteobacteria; Deltaproteobacteria; Desulfuromonadales; Desulfuromonadaceae; Pelobacter

Proteobacteria; Deltaproteobacteria; Desulfuromonadales; Desulfuromonadaceae; Pelobacter

Proteobacteria; Gammaproteobacteria; Vibrionales; Vibrionaceae; unclassified

Proteobacteria; Gammaproteobacteria; Vibrionales; Vibrionaceae; unclassified

Proteobacteria; Gammaproteobacteria; Vibrionales; Vibrionaceae; unclassified

Proteobacteria; Gammaproteobacteria; Vibrionales; Vibrionaceae; unclassified

Proteobacteria; Gammaproteobacteria; Vibrionales; Vibrionaceae; unclassified

Proteobacteria; Gammaproteobacteria; Vibrionales; Vibrionaceae; Vibrio

Proteobacteria; Gammaproteobacteria; Vibrionales; Vibrionaceae; Vibrio

Spirochaetes; Spirochaetes; Spirochaetales; Spirochaetaceae; Spirochaeta

Spirochaetes; Spirochaetes; Spirochaetales; Spirochaetaceae; Spirochaeta

Spirochaetes; Spirochaetes; Spirochaetales; Spirochaetaceae; Spirochaeta

Spirochaetes; Spirochaetes; Spirochaetales; Spirochaetaceae; Spirochaeta

Spirochaetes; Spirochaetes; Spirochaetales; Spirochaetaceae; Spirochaeta

Spirochaetes; Spirochaetes; Spirochaetales; Spirochaetaceae; Spirochaeta

Spirochaetes; Spirochaetes; Spirochaetales; Spirochaetaceae; Spirochaeta

# aerobic_chemoheterotrophy (111 records):

Acidobacteria; Holophagae; Acanthopleuribacterales; Acanthopleuribacteraceae; Acanthopleuribacter

Actinobacteria; Actinobacteria; Actinomycetales; Demequinaceae; Demequina

Bacteroidetes; Flavobacteriia; Flavobacteriales; Cryomorphaceae; Owenweeksia

Bacteroidetes; Flavobacteriia; Flavobacteriales; Flavobacteriaceae; Actibacter

Bacteroidetes; Flavobacteriia; Flavobacteriales; Flavobacteriaceae; Aquimarina

Bacteroidetes; Flavobacteriia; Flavobacteriales; Flavobacteriaceae; Maribacter

Bacteroidetes; Flavobacteriia; Flavobacteriales; Flavobacteriaceae; Maribacter

Bacteroidetes; Flavobacteriia; Flavobacteriales; Flavobacteriaceae; Maribacter

Bacteroidetes; Flavobacteriia; Flavobacteriales; Flavobacteriaceae; Muricauda

Bacteroidetes; Flavobacteriia; Flavobacteriales; Flavobacteriaceae; Muricauda

Bacteroidetes; Flavobacteriia; Flavobacteriales; Flavobacteriaceae; Robiginitalea

Bacteroidetes; Flavobacteriia; Flavobacteriales; Flavobacteriaceae; Tenacibaculum

Bacteroidetes; Flavobacteriia; Flavobacteriales; Flavobacteriaceae; Winogradskyella

Bacteroidetes; Flavobacteriia; Flavobacteriales; Flavobacteriaceae; Winogradskyella

Bacteroidetes; Flavobacteriia; Flavobacteriales; Flavobacteriaceae; Zobellia

Bacteroidetes; Sphingobacteriia; Sphingobacteriales; Saprospiraceae; Lewinella

Bacteroidetes; Sphingobacteriia; Sphingobacteriales; Saprospiraceae; Lewinella

Bacteroidetes; Sphingobacteriia; Sphingobacteriales; Saprospiraceae; Lewinella

Bacteroidetes; Sphingobacteriia; Sphingobacteriales; Saprospiraceae; Lewinella

Bacteroidetes; Sphingobacteriia; Sphingobacteriales; Saprospiraceae; Lewinella

Bacteroidetes; Sphingobacteriia; Sphingobacteriales; Saprospiraceae; Lewinella

Bacteroidetes; Sphingobacteriia; Sphingobacteriales; Saprospiraceae; Lewinella

Proteobacteria; Alphaproteobacteria; Caulobacterales; Hyphomonadaceae; Hyphomonas

Proteobacteria; Alphaproteobacteria; Rhizobiales; Cohaesibacteraceae; Mesorhizobium

Proteobacteria; Alphaproteobacteria; Rhizobiales; Cohaesibacteraceae; Mesorhizobium

Proteobacteria; Alphaproteobacteria; Rhizobiales; Hyphomicrobiaceae; Filomicrobium

Proteobacteria; Alphaproteobacteria; Rhodobacterales; Rhodobacteraceae; Amaricoccus

Proteobacteria; Alphaproteobacteria; Rhodobacterales; Rhodobacteraceae; Citreicella

Proteobacteria; Alphaproteobacteria; Rhodobacterales; Rhodobacteraceae; Jannaschia

Proteobacteria; Alphaproteobacteria; Rhodobacterales; Rhodobacteraceae; Rhodovulum

Proteobacteria; Alphaproteobacteria; Rhodobacterales; Rhodobacteraceae; Rhodovulum

Proteobacteria; Alphaproteobacteria; Rhodobacterales; Rhodobacteraceae; Ruegeria

Proteobacteria; Alphaproteobacteria; Rhodobacterales; Rhodobacteraceae; Ruegeria

Proteobacteria; Alphaproteobacteria; Rhodobacterales; Rhodobacteraceae; Ruegeria

Proteobacteria; Alphaproteobacteria; Rhodobacterales; Rhodobacteraceae; Ruegeria

Proteobacteria; Alphaproteobacteria; Rhodobacterales; Rhodobacteraceae; Stappia

Proteobacteria; Alphaproteobacteria; Rhodobacterales; Rhodobacteraceae; Sulfitobacter

Proteobacteria; Alphaproteobacteria; Rhodobacterales; Rhodobacteraceae; Sulfitobacter

Proteobacteria; Alphaproteobacteria; Rhodospirillales; Rhodospirillaceae; Rhodospirillum

Proteobacteria; Alphaproteobacteria; Rhodospirillales; Rhodospirillaceae; Thalassospira

Proteobacteria; Alphaproteobacteria; Sphingomonadales; Erythrobacteraceae; Altererythrobacter

Proteobacteria; Alphaproteobacteria; Sphingomonadales; Erythrobacteraceae; Altererythrobacter

Proteobacteria; Alphaproteobacteria; Sphingomonadales; Erythrobacteraceae; unclassified

Proteobacteria; Alphaproteobacteria; Sphingomonadales; Erythrobacteraceae; unclassified

Proteobacteria; Alphaproteobacteria; Sphingomonadales; Erythrobacteraceae; unclassified

Proteobacteria; Betaproteobacteria; Burkholderiales; unclassified; Burkholderia

Proteobacteria; Gammaproteobacteria; Alteromonadales; Alteromonadaceae; Agarivorans

Proteobacteria; Gammaproteobacteria; Alteromonadales; Alteromonadaceae; Agarivorans

Proteobacteria; Gammaproteobacteria; Alteromonadales; Alteromonadaceae; Alteromonas

Proteobacteria; Gammaproteobacteria; Alteromonadales; Alteromonadaceae; Alteromonas

Proteobacteria; Gammaproteobacteria; Alteromonadales; Alteromonadaceae; Arenicella

Proteobacteria; Gammaproteobacteria; Alteromonadales; Alteromonadaceae; Arenicella

Proteobacteria; Gammaproteobacteria; Alteromonadales; Alteromonadaceae; Congregibacter

Proteobacteria; Gammaproteobacteria; Alteromonadales; Alteromonadaceae; Congregibacter

Proteobacteria; Gammaproteobacteria; Alteromonadales; Alteromonadaceae; Congregibacter

Proteobacteria; Gammaproteobacteria; Alteromonadales; Alteromonadaceae; Congregibacter

Proteobacteria; Gammaproteobacteria; Alteromonadales; Alteromonadaceae; Haliea

Proteobacteria; Gammaproteobacteria; Alteromonadales; Alteromonadaceae; Haliea

Proteobacteria; Gammaproteobacteria; Alteromonadales; Alteromonadaceae; Microbulbifer

Proteobacteria; Gammaproteobacteria; Alteromonadales; Alteromonadaceae; Saccharophagus

Proteobacteria; Gammaproteobacteria; Alteromonadales; Alteromonadaceae; unclassified

Proteobacteria; Gammaproteobacteria; Alteromonadales; Alteromonadaceae; unclassified

Proteobacteria; Gammaproteobacteria; Alteromonadales; Alteromonadaceae; unclassified

Proteobacteria; Gammaproteobacteria; Alteromonadales; Colwelliaceae; Thalassomonas

Proteobacteria; Gammaproteobacteria; Alteromonadales; Colwelliaceae; Thalassomonas

Proteobacteria; Gammaproteobacteria; Alteromonadales; Pseudoalteromonadaceae; Pseudoalteromonas

Proteobacteria; Gammaproteobacteria; Alteromonadales; Pseudoalteromonadaceae; Pseudoalteromonas

Proteobacteria; Gammaproteobacteria; Alteromonadales; unclassified; unclassified

Proteobacteria; Gammaproteobacteria; Alteromonadales; unclassified_Actinomycetales; unclassified

Proteobacteria; Gammaproteobacteria; Alteromonadales; unclassified_Alteromonadales; unclassified

Proteobacteria; Gammaproteobacteria; Alteromonadales; unclassified_Alteromonadales; unclassified

Proteobacteria; Gammaproteobacteria; Oceanospirillales; Alcanivoracaceae; Marinicella

Proteobacteria; Gammaproteobacteria; Oceanospirillales; Oceanospirillaceae; Amphritea

Proteobacteria; Gammaproteobacteria; Oceanospirillales; Oceanospirillaceae; Amphritea

Proteobacteria; Gammaproteobacteria; Oceanospirillales; Oceanospirillaceae; Amphritea

Proteobacteria; Gammaproteobacteria; Oceanospirillales; Oceanospirillaceae; Marinomonas

Proteobacteria; Gammaproteobacteria; Oceanospirillales; Oceanospirillaceae; Marinomonas

Proteobacteria; Gammaproteobacteria; Oceanospirillales; Oceanospirillaceae; Marinomonas

Proteobacteria; Gammaproteobacteria; Oceanospirillales; Oceanospirillaceae; Marinomonas

Proteobacteria; Gammaproteobacteria; Oceanospirillales; Oceanospirillaceae; Oleibacter

Proteobacteria; Gammaproteobacteria; Oceanospirillales; Oceanospirillaceae; unclassified

Proteobacteria; Gammaproteobacteria; Oceanospirillales; Saccharospirillaceae; Saccharospirillum

Proteobacteria; Gammaproteobacteria; Oceanospirillales; Saccharospirillaceae ; unclassified

Proteobacteria; Gammaproteobacteria; Oceanospirillales; unclassified_Oceanospirillales; unclassified

Proteobacteria; Gammaproteobacteria; Thiotrichales; Piscirickettsiaceae; Cycloclasticus

Proteobacteria; Gammaproteobacteria; Thiotrichales; Piscirickettsiaceae; Cycloclasticus

Proteobacteria; Gammaproteobacteria; Thiotrichales; Piscirickettsiaceae; Cycloclasticus

Proteobacteria; Gammaproteobacteria; Thiotrichales; Thiotrichaceae; Leucothrix

Proteobacteria; Gammaproteobacteria; Vibrionales; Vibrionaceae; unclassified

Proteobacteria; Gammaproteobacteria; Vibrionales; Vibrionaceae; unclassified

Proteobacteria; Gammaproteobacteria; Vibrionales; Vibrionaceae; unclassified

Proteobacteria; Gammaproteobacteria; Vibrionales; Vibrionaceae; unclassified

Proteobacteria; Gammaproteobacteria; Vibrionales; Vibrionaceae; unclassified

Proteobacteria; Gammaproteobacteria; Vibrionales; Vibrionaceae; Vibrio

Proteobacteria; Gammaproteobacteria; Vibrionales; Vibrionaceae; Vibrio

Verrucomicrobia; Verrucomicrobiae; Verrucomicrobiales; Rubritaleaceae; Rubritalea

Verrucomicrobia; Verrucomicrobiae; Verrucomicrobiales; Verrucomicrobiaceae; Haloferula

Verrucomicrobia; Verrucomicrobiae; Verrucomicrobiales; Verrucomicrobiaceae; Haloferula

Verrucomicrobia; Verrucomicrobiae; Verrucomicrobiales; Verrucomicrobiaceae; Luteolibacter

Verrucomicrobia; Verrucomicrobiae; Verrucomicrobiales; Verrucomicrobiaceae; Persicirhabdus

Verrucomicrobia; Verrucomicrobiae; Verrucomicrobiales; Verrucomicrobiaceae; Roseibacillus

Verrucomicrobia; Verrucomicrobiae; Verrucomicrobiales; Verrucomicrobiaceae; Roseibacillus

Verrucomicrobia; Verrucomicrobiae; Verrucomicrobiales; Verrucomicrobiaceae; Roseibacillus

Verrucomicrobia; Verrucomicrobiae; Verrucomicrobiales; Verrucomicrobiaceae; Roseibacillus

Verrucomicrobia; Verrucomicrobiae; Verrucomicrobiales; Verrucomicrobiaceae; unclassified

Verrucomicrobia; Verrucomicrobiae; Verrucomicrobiales; Verrucomicrobiaceae; unclassified

Verrucomicrobia; Verrucomicrobiae; Verrucomicrobiales; Verrucomicrobiaceae; unclassified

Verrucomicrobia; Verrucomicrobiae; Verrucomicrobiales; Verrucomicrobiaceae; unclassified

Verrucomicrobia; Verrucomicrobiae; Verrucomicrobiales; Verrucomicrobiaceae; unclassified

Verrucomicrobia; Verrucomicrobiae; Verrucomicrobiales; Verrucomicrobiaceae; unclassified

Verrucomicrobia; Verrucomicrobiae; Verrucomicrobiales; Verrucomicrobiaceae; unclassified

# invertebrate_parasites (0 records):

# human_pathogens_septicemia (0 records)

# human_pathogens_pneumonia (0 records):

# human_pathogens_nosocomia (0 records):

# human_pathogens_meningitis (0 records):

# human_pathogens_gastroenteritis (0 records):

# human_pathogens_diarrhea (0 records):

# human_pathogens_all (0 records):

# fish_parasites (0 records):

# human_gut (0 records):

# human_associated (0 records):

# mammal_gut (0 records):

# animal_parasites_or_symbionts (0 records):

# plant_pathogen (0 records):

# oil_bioremediation (0 records):

# aromatic_hydrocarbon_degradation (3 records):

Proteobacteria; Gammaproteobacteria; Thiotrichales; Piscirickettsiaceae; Cycloclasticus

Proteobacteria; Gammaproteobacteria; Thiotrichales; Piscirickettsiaceae; Cycloclasticus

Proteobacteria; Gammaproteobacteria; Thiotrichales; Piscirickettsiaceae; Cycloclasticus

# aromatic_compound_degradation (6 records):

Firmicutes; Clostridia; Clostridiales; Lachnospiraceae; Sporobacterium

Firmicutes; Clostridia; Clostridiales; Lachnospiraceae; Sporobacterium

Proteobacteria; Gammaproteobacteria; Alteromonadales; Alteromonadaceae; Microbulbifer

Proteobacteria; Gammaproteobacteria; Thiotrichales; Piscirickettsiaceae; Cycloclasticus

Proteobacteria; Gammaproteobacteria; Thiotrichales; Piscirickettsiaceae; Cycloclasticus

Proteobacteria; Gammaproteobacteria; Thiotrichales; Piscirickettsiaceae; Cycloclasticus

# aliphatic_non_methane_hydrocarbon_degradation (0 records):

# hydrocarbon_degradation (5 records):

Proteobacteria; Alphaproteobacteria; Rhizobiales; Methylocystaceae; Methylocystis

Proteobacteria; Alphaproteobacteria; Rhizobiales; Methylocystaceae; Terasakiella

Proteobacteria; Gammaproteobacteria; Thiotrichales; Piscirickettsiaceae; Cycloclasticus

Proteobacteria; Gammaproteobacteria; Thiotrichales; Piscirickettsiaceae; Cycloclasticus

Proteobacteria; Gammaproteobacteria; Thiotrichales; Piscirickettsiaceae; Cycloclasticus

# dark_iron_oxidation (0 records):

# iron_respiration (0 records):

# nitrate_respiration (0 records):

# nitrate_reduction (2 records):

Proteobacteria; Gammaproteobacteria; Vibrionales; Vibrionaceae; Vibrio

Proteobacteria; Gammaproteobacteria; Vibrionales; Vibrionaceae; Vibrio

# nitrogen_respiration (0 records):

# fumarate_respiration (0 records):

# intracellular_parasites (2 records):

Proteobacteria; Alphaproteobacteria; Rickettsiales; Midichloriaceae; unclassified

Proteobacteria; Alphaproteobacteria; Rickettsiales; Rickettsiaceae; unclassified

# chlorate_reducers (0 records):

# predatory_or_exoparasitic (2 records):

Proteobacteria; Alphaproteobacteria; Bdellovibrionales; Bdellovibrionaceae; unclassified

Proteobacteria; Oligoflexia; Bdellovibrionales; Bdellovibrionaceae; unclassified

# chloroplasts (0 records):

# nonphotosynthetic_cyanobacteria (0 records):

# photosynthetic_cyanobacteria (0 records):

# anoxygenic_photoautotrophy_H2_oxidizing (0 records):

# anoxygenic_photoautotrophy_S_oxidizing (8 records):

Proteobacteria; Gammaproteobacteria; Chromatiales; Chromatiaceae; Thiohalocapsa

Proteobacteria; Gammaproteobacteria; Chromatiales; Chromatiaceae; Thiohalocapsa

Proteobacteria; Gammaproteobacteria; Chromatiales; Ectothiorhodospiraceae; Thioalkalivibrio

Proteobacteria; Gammaproteobacteria; Chromatiales; Ectothiorhodospiraceae; unclassified

Proteobacteria; Gammaproteobacteria; Chromatiales; Ectothiorhodospiraceae; unclassified

Proteobacteria; Gammaproteobacteria; Chromatiales; Ectothiorhodospiraceae; unclassified

Proteobacteria; Gammaproteobacteria; Chromatiales; Ectothiorhodospiraceae; unclassified

Proteobacteria; Gammaproteobacteria; Chromatiales; Ectothiorhodospiraceae; unclassified

# anoxygenic_photoautotrophy_Fe_oxidizing (0 records):

# anoxygenic_photoautotrophy (10 records):

Proteobacteria; Alphaproteobacteria; Rhodobacterales; Rhodobacteraceae; Rhodovulum

Proteobacteria; Alphaproteobacteria; Rhodobacterales; Rhodobacteraceae; Rhodovulum

Proteobacteria; Gammaproteobacteria; Chromatiales; Chromatiaceae; Thiohalocapsa

Proteobacteria; Gammaproteobacteria; Chromatiales; Chromatiaceae; Thiohalocapsa

Proteobacteria; Gammaproteobacteria; Chromatiales; Ectothiorhodospiraceae; Thioalkalivibrio

Proteobacteria; Gammaproteobacteria; Chromatiales; Ectothiorhodospiraceae; unclassified

Proteobacteria; Gammaproteobacteria; Chromatiales; Ectothiorhodospiraceae; unclassified

Proteobacteria; Gammaproteobacteria; Chromatiales; Ectothiorhodospiraceae; unclassified

Proteobacteria; Gammaproteobacteria; Chromatiales; Ectothiorhodospiraceae; unclassified

Proteobacteria; Gammaproteobacteria; Chromatiales; Ectothiorhodospiraceae; unclassified

# oxygenic_photoautotrophy (0 records):

# photoautotrophy (10 records):

Proteobacteria; Alphaproteobacteria; Rhodobacterales; Rhodobacteraceae; Rhodovulum

Proteobacteria; Alphaproteobacteria; Rhodobacterales; Rhodobacteraceae; Rhodovulum

Proteobacteria; Gammaproteobacteria; Chromatiales; Chromatiaceae; Thiohalocapsa

Proteobacteria; Gammaproteobacteria; Chromatiales; Chromatiaceae; Thiohalocapsa

Proteobacteria; Gammaproteobacteria; Chromatiales; Ectothiorhodospiraceae; Thioalkalivibrio

Proteobacteria; Gammaproteobacteria; Chromatiales; Ectothiorhodospiraceae; unclassified

Proteobacteria; Gammaproteobacteria; Chromatiales; Ectothiorhodospiraceae; unclassified

Proteobacteria; Gammaproteobacteria; Chromatiales; Ectothiorhodospiraceae; unclassified

Proteobacteria; Gammaproteobacteria; Chromatiales; Ectothiorhodospiraceae; unclassified

Proteobacteria; Gammaproteobacteria; Chromatiales; Ectothiorhodospiraceae; unclassified

# aerobic_anoxygenic_phototrophy (0 records):

# photoheterotrophy (3 records):

Proteobacteria; Alphaproteobacteria; Rhodobacterales; Rhodobacteraceae; Rhodovulum

Proteobacteria; Alphaproteobacteria; Rhodobacterales; Rhodobacteraceae; Rhodovulum

Proteobacteria; Alphaproteobacteria; Rhodospirillales; Rhodospirillaceae; Rhodospirillum

# phototrophy (11 records):

Proteobacteria; Alphaproteobacteria; Rhodobacterales; Rhodobacteraceae; Rhodovulum

Proteobacteria; Alphaproteobacteria; Rhodobacterales; Rhodobacteraceae; Rhodovulum

Proteobacteria; Alphaproteobacteria; Rhodospirillales; Rhodospirillaceae; Rhodospirillum

Proteobacteria; Gammaproteobacteria; Chromatiales; Chromatiaceae; Thiohalocapsa

Proteobacteria; Gammaproteobacteria; Chromatiales; Chromatiaceae; Thiohalocapsa

Proteobacteria; Gammaproteobacteria; Chromatiales; Ectothiorhodospiraceae; Thioalkalivibrio

Proteobacteria; Gammaproteobacteria; Chromatiales; Ectothiorhodospiraceae; unclassified

Proteobacteria; Gammaproteobacteria; Chromatiales; Ectothiorhodospiraceae; unclassified

Proteobacteria; Gammaproteobacteria; Chromatiales; Ectothiorhodospiraceae; unclassified

Proteobacteria; Gammaproteobacteria; Chromatiales; Ectothiorhodospiraceae; unclassified

Proteobacteria; Gammaproteobacteria; Chromatiales; Ectothiorhodospiraceae; unclassified

# plastic_degradation (0 records):

# ureolysis (2 records):

Proteobacteria; Alphaproteobacteria; Rhizobiales; Cohaesibacteraceae; Mesorhizobium

Proteobacteria; Alphaproteobacteria; Rhizobiales; Cohaesibacteraceae; Mesorhizobium

# reductive_acetogenesis (0 records):

# chemoheterotrophy (148 records):

Acidobacteria; Holophagae; Acanthopleuribacterales; Acanthopleuribacteraceae; Acanthopleuribacter

Actinobacteria; Actinobacteria; Actinomycetales; Demequinaceae; Demequina

Actinobacteria; Actinobacteria; Actinomycetales; Propionibacteriaceae; Propionibacterium

Bacteroidetes; Flavobacteriia; Flavobacteriales; Cryomorphaceae; Owenweeksia

Bacteroidetes; Flavobacteriia; Flavobacteriales; Flavobacteriaceae; Actibacter

Bacteroidetes; Flavobacteriia; Flavobacteriales; Flavobacteriaceae; Aquimarina

Bacteroidetes; Flavobacteriia; Flavobacteriales; Flavobacteriaceae; Maribacter

Bacteroidetes; Flavobacteriia; Flavobacteriales; Flavobacteriaceae; Maribacter

Bacteroidetes; Flavobacteriia; Flavobacteriales; Flavobacteriaceae; Maribacter

Bacteroidetes; Flavobacteriia; Flavobacteriales; Flavobacteriaceae; Muricauda

Bacteroidetes; Flavobacteriia; Flavobacteriales; Flavobacteriaceae; Muricauda

Bacteroidetes; Flavobacteriia; Flavobacteriales; Flavobacteriaceae; Robiginitalea

Bacteroidetes; Flavobacteriia; Flavobacteriales; Flavobacteriaceae; Tenacibaculum

Bacteroidetes; Flavobacteriia; Flavobacteriales; Flavobacteriaceae; Winogradskyella

Bacteroidetes; Flavobacteriia; Flavobacteriales; Flavobacteriaceae; Winogradskyella

Bacteroidetes; Flavobacteriia; Flavobacteriales; Flavobacteriaceae; Zobellia

Bacteroidetes; Sphingobacteriia; Sphingobacteriales; Saprospiraceae; Lewinella

Bacteroidetes; Sphingobacteriia; Sphingobacteriales; Saprospiraceae; Lewinella

Bacteroidetes; Sphingobacteriia; Sphingobacteriales; Saprospiraceae; Lewinella

Bacteroidetes; Sphingobacteriia; Sphingobacteriales; Saprospiraceae; Lewinella

Bacteroidetes; Sphingobacteriia; Sphingobacteriales; Saprospiraceae; Lewinella

Bacteroidetes; Sphingobacteriia; Sphingobacteriales; Saprospiraceae; Lewinella

Bacteroidetes; Sphingobacteriia; Sphingobacteriales; Saprospiraceae; Lewinella

Fibrobacteres; Fibrobacteria; Fibrobacterales; Fibrobacteraceae; Fibrobacter

Firmicutes; Clostridia; Clostridiales; Lachnospiraceae; Sporobacterium

Firmicutes; Clostridia; Clostridiales; Lachnospiraceae; Sporobacterium

Proteobacteria; Alphaproteobacteria; Caulobacterales; Hyphomonadaceae; Hyphomonas

Proteobacteria; Alphaproteobacteria; Rhizobiales; Cohaesibacteraceae; Mesorhizobium

Proteobacteria; Alphaproteobacteria; Rhizobiales; Cohaesibacteraceae; Mesorhizobium

Proteobacteria; Alphaproteobacteria; Rhizobiales; Hyphomicrobiaceae; Filomicrobium

Proteobacteria; Alphaproteobacteria; Rhizobiales; Methylocystaceae; Methylocystis

Proteobacteria; Alphaproteobacteria; Rhizobiales; Methylocystaceae; Terasakiella

Proteobacteria; Alphaproteobacteria; Rhodobacterales; Rhodobacteraceae; Amaricoccus

Proteobacteria; Alphaproteobacteria; Rhodobacterales; Rhodobacteraceae; Citreicella

Proteobacteria; Alphaproteobacteria; Rhodobacterales; Rhodobacteraceae; Jannaschia

Proteobacteria; Alphaproteobacteria; Rhodobacterales; Rhodobacteraceae; Rhodovulum

Proteobacteria; Alphaproteobacteria; Rhodobacterales; Rhodobacteraceae; Rhodovulum

Proteobacteria; Alphaproteobacteria; Rhodobacterales; Rhodobacteraceae; Ruegeria

Proteobacteria; Alphaproteobacteria; Rhodobacterales; Rhodobacteraceae; Ruegeria

Proteobacteria; Alphaproteobacteria; Rhodobacterales; Rhodobacteraceae; Ruegeria

Proteobacteria; Alphaproteobacteria; Rhodobacterales; Rhodobacteraceae; Ruegeria

Proteobacteria; Alphaproteobacteria; Rhodobacterales; Rhodobacteraceae; Stappia

Proteobacteria; Alphaproteobacteria; Rhodobacterales; Rhodobacteraceae; Sulfitobacter

Proteobacteria; Alphaproteobacteria; Rhodobacterales; Rhodobacteraceae; Sulfitobacter

Proteobacteria; Alphaproteobacteria; Rhodospirillales; Rhodospirillaceae; Rhodospirillum

Proteobacteria; Alphaproteobacteria; Rhodospirillales; Rhodospirillaceae; Thalassospira

Proteobacteria; Alphaproteobacteria; Sphingomonadales; Erythrobacteraceae; Altererythrobacter

Proteobacteria; Alphaproteobacteria; Sphingomonadales; Erythrobacteraceae; Altererythrobacter

Proteobacteria; Alphaproteobacteria; Sphingomonadales; Erythrobacteraceae; unclassified

Proteobacteria; Alphaproteobacteria; Sphingomonadales; Erythrobacteraceae; unclassified

Proteobacteria; Alphaproteobacteria; Sphingomonadales; Erythrobacteraceae; unclassified

Proteobacteria; Betaproteobacteria; Burkholderiales; unclassified; Burkholderia

Proteobacteria; Betaproteobacteria; Methylophilales; Methylophilaceae; unclassified

Proteobacteria; Betaproteobacteria; Methylophilales; Methylophilaceae; unclassified

Proteobacteria; Betaproteobacteria; Methylophilales; Methylophilaceae; unclassified

Proteobacteria; Betaproteobacteria; Methylophilales; Methylophilaceae; unclassified

Proteobacteria; Betaproteobacteria; Methylophilales; Methylophilaceae; unclassified

Proteobacteria; Deltaproteobacteria; Desulfobacterales; Desulfobacteraceae; Desulfobacterium

Proteobacteria; Deltaproteobacteria; Desulfobacterales; Desulfobulbaceae; Desulfobulbus

Proteobacteria; Deltaproteobacteria; Desulfobacterales; Desulfobulbaceae; Desulfobulbus

Proteobacteria; Deltaproteobacteria; Desulfobacterales; Desulfobulbaceae; Desulfobulbus

Proteobacteria; Deltaproteobacteria; Desulfobacterales; Desulfobulbaceae; Desulfobulbus

Proteobacteria; Deltaproteobacteria; Desulfobacterales; Desulfobulbaceae; Desulfobulbus

Proteobacteria; Deltaproteobacteria; Desulfobacterales; Desulfobulbaceae; Desulfobulbus

Proteobacteria; Deltaproteobacteria; Desulfobacterales; Desulfobulbaceae; Desulfobulbus

Proteobacteria; Deltaproteobacteria; Desulfobacterales; Desulfobulbaceae; Desulfobulbus

Proteobacteria; Deltaproteobacteria; Desulfobacterales; Desulfobulbaceae; Desulforhopalus

Proteobacteria; Deltaproteobacteria; Desulfobacterales; Desulfobulbaceae; Desulforhopalus

Proteobacteria; Deltaproteobacteria; Desulfovibrionales; Desulfovibrionaceae; Desulfocurvus

Proteobacteria; Deltaproteobacteria; Desulfuromonadales; Desulfuromonadaceae; Malonomonas

Proteobacteria; Deltaproteobacteria; Desulfuromonadales; Desulfuromonadaceae; Pelobacter

Proteobacteria; Deltaproteobacteria; Desulfuromonadales; Desulfuromonadaceae; Pelobacter

Proteobacteria; Gammaproteobacteria; Alteromonadales; Alteromonadaceae; Agarivorans

Proteobacteria; Gammaproteobacteria; Alteromonadales; Alteromonadaceae; Agarivorans

Proteobacteria; Gammaproteobacteria; Alteromonadales; Alteromonadaceae; Alteromonas

Proteobacteria; Gammaproteobacteria; Alteromonadales; Alteromonadaceae; Alteromonas

Proteobacteria; Gammaproteobacteria; Alteromonadales; Alteromonadaceae; Arenicella

Proteobacteria; Gammaproteobacteria; Alteromonadales; Alteromonadaceae; Arenicella

Proteobacteria; Gammaproteobacteria; Alteromonadales; Alteromonadaceae; Congregibacter

Proteobacteria; Gammaproteobacteria; Alteromonadales; Alteromonadaceae; Congregibacter

Proteobacteria; Gammaproteobacteria; Alteromonadales; Alteromonadaceae; Congregibacter

Proteobacteria; Gammaproteobacteria; Alteromonadales; Alteromonadaceae; Congregibacter

Proteobacteria; Gammaproteobacteria; Alteromonadales; Alteromonadaceae; Haliea

Proteobacteria; Gammaproteobacteria; Alteromonadales; Alteromonadaceae; Haliea

Proteobacteria; Gammaproteobacteria; Alteromonadales; Alteromonadaceae; Microbulbifer

Proteobacteria; Gammaproteobacteria; Alteromonadales; Alteromonadaceae; Saccharophagus

Proteobacteria; Gammaproteobacteria; Alteromonadales; Alteromonadaceae; unclassified

Proteobacteria; Gammaproteobacteria; Alteromonadales; Alteromonadaceae; unclassified

Proteobacteria; Gammaproteobacteria; Alteromonadales; Alteromonadaceae; unclassified

Proteobacteria; Gammaproteobacteria; Alteromonadales; Colwelliaceae; Thalassomonas

Proteobacteria; Gammaproteobacteria; Alteromonadales; Colwelliaceae; Thalassomonas

Proteobacteria; Gammaproteobacteria; Alteromonadales; Pseudoalteromonadaceae; Pseudoalteromonas

Proteobacteria; Gammaproteobacteria; Alteromonadales; Pseudoalteromonadaceae; Pseudoalteromonas

Proteobacteria; Gammaproteobacteria; Alteromonadales; unclassified; unclassified

Proteobacteria; Gammaproteobacteria; Alteromonadales; unclassified_Actinomycetales; unclassified

Proteobacteria; Gammaproteobacteria; Alteromonadales; unclassified_Alteromonadales; unclassified

Proteobacteria; Gammaproteobacteria; Alteromonadales; unclassified_Alteromonadales; unclassified

Proteobacteria; Gammaproteobacteria; Oceanospirillales; Alcanivoracaceae; Marinicella

Proteobacteria; Gammaproteobacteria; Oceanospirillales; Oceanospirillaceae; Amphritea

Proteobacteria; Gammaproteobacteria; Oceanospirillales; Oceanospirillaceae; Amphritea

Proteobacteria; Gammaproteobacteria; Oceanospirillales; Oceanospirillaceae; Amphritea

Proteobacteria; Gammaproteobacteria; Oceanospirillales; Oceanospirillaceae; Marinomonas

Proteobacteria; Gammaproteobacteria; Oceanospirillales; Oceanospirillaceae; Marinomonas

Proteobacteria; Gammaproteobacteria; Oceanospirillales; Oceanospirillaceae; Marinomonas

Proteobacteria; Gammaproteobacteria; Oceanospirillales; Oceanospirillaceae; Marinomonas

Proteobacteria; Gammaproteobacteria; Oceanospirillales; Oceanospirillaceae; Oleibacter

Proteobacteria; Gammaproteobacteria; Oceanospirillales; Oceanospirillaceae; unclassified

Proteobacteria; Gammaproteobacteria; Oceanospirillales; Saccharospirillaceae; Saccharospirillum

Proteobacteria; Gammaproteobacteria; Oceanospirillales; Saccharospirillaceae ; unclassified

Proteobacteria; Gammaproteobacteria; Oceanospirillales; unclassified_Oceanospirillales; unclassified

Proteobacteria; Gammaproteobacteria; Thiotrichales; Piscirickettsiaceae; Cycloclasticus

Proteobacteria; Gammaproteobacteria; Thiotrichales; Piscirickettsiaceae; Cycloclasticus

Proteobacteria; Gammaproteobacteria; Thiotrichales; Piscirickettsiaceae; Cycloclasticus

Proteobacteria; Gammaproteobacteria; Thiotrichales; Piscirickettsiaceae; Methylophaga

Proteobacteria; Gammaproteobacteria; Thiotrichales; Piscirickettsiaceae; Methylophaga

Proteobacteria; Gammaproteobacteria; Thiotrichales; Piscirickettsiaceae; Methylophaga

Proteobacteria; Gammaproteobacteria; Thiotrichales; Piscirickettsiaceae; Methylophaga

Proteobacteria; Gammaproteobacteria; Thiotrichales; Thiotrichaceae; Leucothrix

Proteobacteria; Gammaproteobacteria; Vibrionales; Vibrionaceae; unclassified

Proteobacteria; Gammaproteobacteria; Vibrionales; Vibrionaceae; unclassified

Proteobacteria; Gammaproteobacteria; Vibrionales; Vibrionaceae; unclassified

Proteobacteria; Gammaproteobacteria; Vibrionales; Vibrionaceae; unclassified

Proteobacteria; Gammaproteobacteria; Vibrionales; Vibrionaceae; unclassified

Proteobacteria; Gammaproteobacteria; Vibrionales; Vibrionaceae; Vibrio

Proteobacteria; Gammaproteobacteria; Vibrionales; Vibrionaceae; Vibrio

Spirochaetes; Spirochaetes; Spirochaetales; Spirochaetaceae; Spirochaeta

Spirochaetes; Spirochaetes; Spirochaetales; Spirochaetaceae; Spirochaeta

Spirochaetes; Spirochaetes; Spirochaetales; Spirochaetaceae; Spirochaeta

Spirochaetes; Spirochaetes; Spirochaetales; Spirochaetaceae; Spirochaeta

Spirochaetes; Spirochaetes; Spirochaetales; Spirochaetaceae; Spirochaeta

Spirochaetes; Spirochaetes; Spirochaetales; Spirochaetaceae; Spirochaeta

Spirochaetes; Spirochaetes; Spirochaetales; Spirochaetaceae; Spirochaeta

Verrucomicrobia; Verrucomicrobiae; Verrucomicrobiales; Rubritaleaceae; Rubritalea

Verrucomicrobia; Verrucomicrobiae; Verrucomicrobiales; Verrucomicrobiaceae; Haloferula

Verrucomicrobia; Verrucomicrobiae; Verrucomicrobiales; Verrucomicrobiaceae; Haloferula

Verrucomicrobia; Verrucomicrobiae; Verrucomicrobiales; Verrucomicrobiaceae; Luteolibacter

Verrucomicrobia; Verrucomicrobiae; Verrucomicrobiales; Verrucomicrobiaceae; Persicirhabdus

Verrucomicrobia; Verrucomicrobiae; Verrucomicrobiales; Verrucomicrobiaceae; Roseibacillus

Verrucomicrobia; Verrucomicrobiae; Verrucomicrobiales; Verrucomicrobiaceae; Roseibacillus

Verrucomicrobia; Verrucomicrobiae; Verrucomicrobiales; Verrucomicrobiaceae; Roseibacillus

Verrucomicrobia; Verrucomicrobiae; Verrucomicrobiales; Verrucomicrobiaceae; Roseibacillus

Verrucomicrobia; Verrucomicrobiae; Verrucomicrobiales; Verrucomicrobiaceae; unclassified

Verrucomicrobia; Verrucomicrobiae; Verrucomicrobiales; Verrucomicrobiaceae; unclassified

Verrucomicrobia; Verrucomicrobiae; Verrucomicrobiales; Verrucomicrobiaceae; unclassified

Verrucomicrobia; Verrucomicrobiae; Verrucomicrobiales; Verrucomicrobiaceae; unclassified

Verrucomicrobia; Verrucomicrobiae; Verrucomicrobiales; Verrucomicrobiaceae; unclassified

Verrucomicrobia; Verrucomicrobiae; Verrucomicrobiales; Verrucomicrobiaceae; unclassified

Verrucomicrobia; Verrucomicrobiae; Verrucomicrobiales; Verrucomicrobiaceae; unclassified
