# Supplementary material for: Distinct Endophytic Bacterial Communities Inhabiting Seagrass Seeds
Source: Front Microbiol. 2021 Sep 21;12:703014. doi: 10.3389/fmicb.2021.703014 (PMC8491609; doi:10.3389/fmicb.2021.703014)
Supplement: Supplementary file 2 [file Table_2.DOCX]

**Supplementary Table2**. Number of OTUs reported for the classes of the five most abundant phyla present within *H. ovalis* microenvironments.

| Phylum | Class | Number of OTUs | Total per phylum |
| --- | --- | --- | --- |
| Acidobacteria | Acidobacteriia | 27 | 31 |
|  | Holophagae | 4 |  |
| Actinobacteria | Acidimicrobiia | 16 | 46 |
|  | Actinobacteria | 27 |  |
|  | Coriobacteriia | 1 |  |
|  | Thermoleophilia | 2 |  |
| Bacteroidetes | Bacteroidia | 15 | 140 |
|  | Cytophagia | 6 |  |
|  | Flavobacteriia | 55 |  |
|  | Sphingobacteriia | 45 |  |
|  | Unclassified | 19 |  |
| Firmicutes | Bacili | 2 | 17 |
|  | Clostridia | 15 |  |
| Proteobacteria | Alphaproteobacteria | 97 | 426 |
|  | Betaproteobacteria | 13 |  |
|  | Deltaproteobacteria | 131 |  |
|  | Epsilonproteobacteria | 9 |  |
|  | Gammaproteobacteria | 173 |  |
|  | Oligoflexia | 3 |  |
