# Supplementary material for: Distinct Endophytic Bacterial Communities Inhabiting Seagrass Seeds
Source: Front Microbiol. 2021 Sep 21;12:703014. doi: 10.3389/fmicb.2021.703014 (PMC8491609; doi:10.3389/fmicb.2021.703014)
Supplement: Supplementary file 3 [file Table_3.DOCX]

**Supplementary Tble3.** (A) Number of observed OTUs (richness) and Shannon index (diversity) for each replicate of the microenvironments and (B) mean values of the number of observed OTUs and Shannon diversity index per microenvironment shown with the standard error.

A

| Tissue | Samples | Observed | Shannon |
| --- | --- | --- | --- |
| Flower | FlR1 | 351 | 4.593 |
|  | FlR2 | 319 | 4.664 |
|  | FlR3 | 232 | 3.902 |
|  | FlR4 | 394 | 3.971 |
|  | FlR5 | 328 | 4.040 |
| Fruit | FrR1 | 389 | 4.464 |
|  | FrR2 | 290 | 3.954 |
|  | FrR3 | 339 | 3.881 |
|  | FrR4 | 404 | 4.428 |
|  | FrR5 | 346 | 4.340 |
| Leaf | LwFlR1 | 335 | 3.873 |
|  | LwFlR2 | 282 | 3.772 |
|  | LwFlR3 | 314 | 3.557 |
|  | LwFlR4 | 307 | 3.654 |
|  | LwFlR5 | 223 | 3.016 |
|  | LwFrR1 | 284 | 3.920 |
|  | LwFrR2 | 312 | 3.632 |
|  | LwFrR3 | 285 | 4.020 |
|  | LwFrR4 | 247 | 3.922 |
|  | LwFrR5 | 355 | 3.865 |
| Root | RwFlR1 | 326 | 4.048 |
|  | RwFlR2 | 329 | 4.175 |
|  | RwFlR3 | 414 | 4.330 |
|  | RwFlR4 | 372 | 3.854 |
|  | RwFlR5 | 391 | 4.048 |
|  | RwFrR1 | 298 | 3.835 |
|  | RwFrR2 | 337 | 3.963 |
|  | RwFrR3 | 316 | 3.838 |
|  | RwFrR4 | 392 | 3.816 |
|  | RwFrR5 | 374 | 3.731 |
| Seed | SeedR1 | 70 | 1.736 |
|  | SeedR2 | 54 | 3.191 |
|  | SeedR3 | 52 | 1.318 |
|  | SeedR4 | 234 | 3.014 |
|  | SeedR5 | 33 | 3.231 |
| Sediment | SwFlR1 | 374 | 4.749 |
|  | SwFlR2 | 390 | 4.738 |
|  | SwFlR3 | 375 | 4.716 |
|  | SwFlR4 | 445 | 4.864 |
|  | SwFlR5 | 399 | 4.723 |
|  | SwFrR1 | 407 | 4.845 |
|  | SwFrR2 | 391 | 4.734 |
|  | SwFrR3 | 414 | 4.862 |
|  | SwFrR4 | 400 | 4.716 |
|  | SwFrR5 | 411 | 4.735 |

B

| **Tissue** | **Observed** | **Shannon** |
| --- | --- | --- |
| **Flower** | 324.8 (± 26.58) | 4.23 (± 0.16) |
| **Fruit** | 353.6 (± 20.14) | 4.21(± 0.12) |
| **Leaf** | 294.4 (± 12.41) | 3.72 (± 0.09) |
| **Root** | 354.9 (± 12.20) | 3.96 (± 0.06) |
| **Seed** | 88.6 (± 36.82) | 2.50 (± 0.40) |
| **Sediment** | 400.6 (± 6.56) | 4.77 (± 0.019) |
