# Supplementary material for: Distinct Endophytic Bacterial Communities Inhabiting Seagrass Seeds
Source: Front Microbiol. 2021 Sep 21;12:703014. doi: 10.3389/fmicb.2021.703014 (PMC8491609; doi:10.3389/fmicb.2021.703014)
Supplement: Supplementary file 4 [file Table_4.DOCX]

**Supplementary Tble4.** PERMANOVA test performed using the *adonis* function from the *vegan* package in R with 999 permutations to test hypotheses about differences in the bacterial assemblage among microenvironments
