# Supplementary material for: Distinct Endophytic Bacterial Communities Inhabiting Seagrass Seeds
Source: Front Microbiol. 2021 Sep 21;12:703014. doi: 10.3389/fmicb.2021.703014 (PMC8491609; doi:10.3389/fmicb.2021.703014)
Supplement: Supplementary file 5 [file Table_5.DOCX]

**Supplementary Table5**. (A) Results from the SIMPER analysis. The two OTUs that contribute the most to the difference between pairwise test are shown together with the contribution to average between-group dissimilarity and the ordered cumulative contribution. (B) The taxonomy of the OTUs are reported, “-“ stands for same as above.

A

| Tissue | OTU | Average | Cumsum |
| --- | --- | --- | --- |
| flower - fruit | Otu348 | 0.00310834 | 0.00669909 |
|  | Otu616 | 0.00243687 | 0.01195103 |
|  |  |  |  |
| flower - leaf | Otu348 | 0.00348471 | 0.00723599 |
|  | Otu22 | 0.00327578 | 0.01403814 |
|  |  |  |  |
| flower - root | Otu28 | 0.003204241 | 0.006389387 |
|  | Otu348 | 0.003016464 | 0.012404338 |
|  |  |  |  |
| flower - seed | Otu348 | 0.00519721 | 0.006693208 |
|  | Otu28 | 0.003959386 | 0.011792289 |
|  |  |  |  |
| flower - sediment | Otu28 | 0.002800375 | 0.005679061 |
|  | Otu348 | 0.002791144 | 0.0113394 |
|  |  |  |  |
| fruit - leaf | Otu96 | 0.003521977 | 0.006765359 |
|  | Otu12 | 0.003488167 | 0.013465771 |
|  |  |  |  |
| fruit - root | Otu818 | 0.00270266 | 0.005662744 |
|  | Otu252 | 0.002559261 | 0.011025031 |
|  |  |  |  |
| fruit - seed | Otu4 | 0.004004376 | 0.005653957 |
|  | Otu53 | 0.003939754 | 0.011216671 |
|  |  |  |  |
| fruit - sediment | Otu22 | 0.0026592 | 0.0043256 |
|  | Otu38 | 0.00253514 | 0.00844941 |
|  |  |  |  |
| leaf - root | Otu2 | 0.003875247 | 0.00584625 |
|  | Otu27 | 0.003497041 | 0.01112193 |
|  |  |  |  |
| leaf - seed | Otu5 | 0.006545314 | 0.007770912 |
|  | Otu153 | 0.006421087 | 0.015394335 |
|  |  |  |  |
| leaf - sediment | Otu53 | 0.003377125 | 0.005345329 |
|  | Otu153 | 0.003088402 | 0.010233665 |
|  |  |  |  |
| root - seed | Otu252 | 0.004385573 | 0.005600761 |
|  | Otu1017 | 0.004296338 | 0.011087562 |
|  |  |  |  |
| root - sediment | Otu2 | 0.002700268 | 0.004875109 |
|  | Otu27 | 0.002564393 | 0.009504908 |
|  |  |  |  |
| seed - sediment | Otu14 | 0.004381066 | 0.00498451 |
|  | Otu3 | 0.004375809 | 0.009963039 |

B
