# Supplementary material for: Distinct Endophytic Bacterial Communities Inhabiting Seagrass Seeds
Source: Front Microbiol. 2021 Sep 21;12:703014. doi: 10.3389/fmicb.2021.703014 (PMC8491609; doi:10.3389/fmicb.2021.703014)
Supplement: Supplementary file 6 [file Table_6.DOCX]

**Supplementary Table6.** Relative abundance of the families present in the core microbiome of *H. ovalis* rhizospheric sediment, roots, leaves, flowers, fruits and seeds, expressed as percentage. Only the families for which the relative abundance was higher than 0.1 are shown.

,
