# Supplementary material for: Distinct Endophytic Bacterial Communities Inhabiting Seagrass Seeds
Source: Front Microbiol. 2021 Sep 21;12:703014. doi: 10.3389/fmicb.2021.703014 (PMC8491609; doi:10.3389/fmicb.2021.703014)
Supplement: Supplementary file 7 [file Data_Sheet_1.PDF]

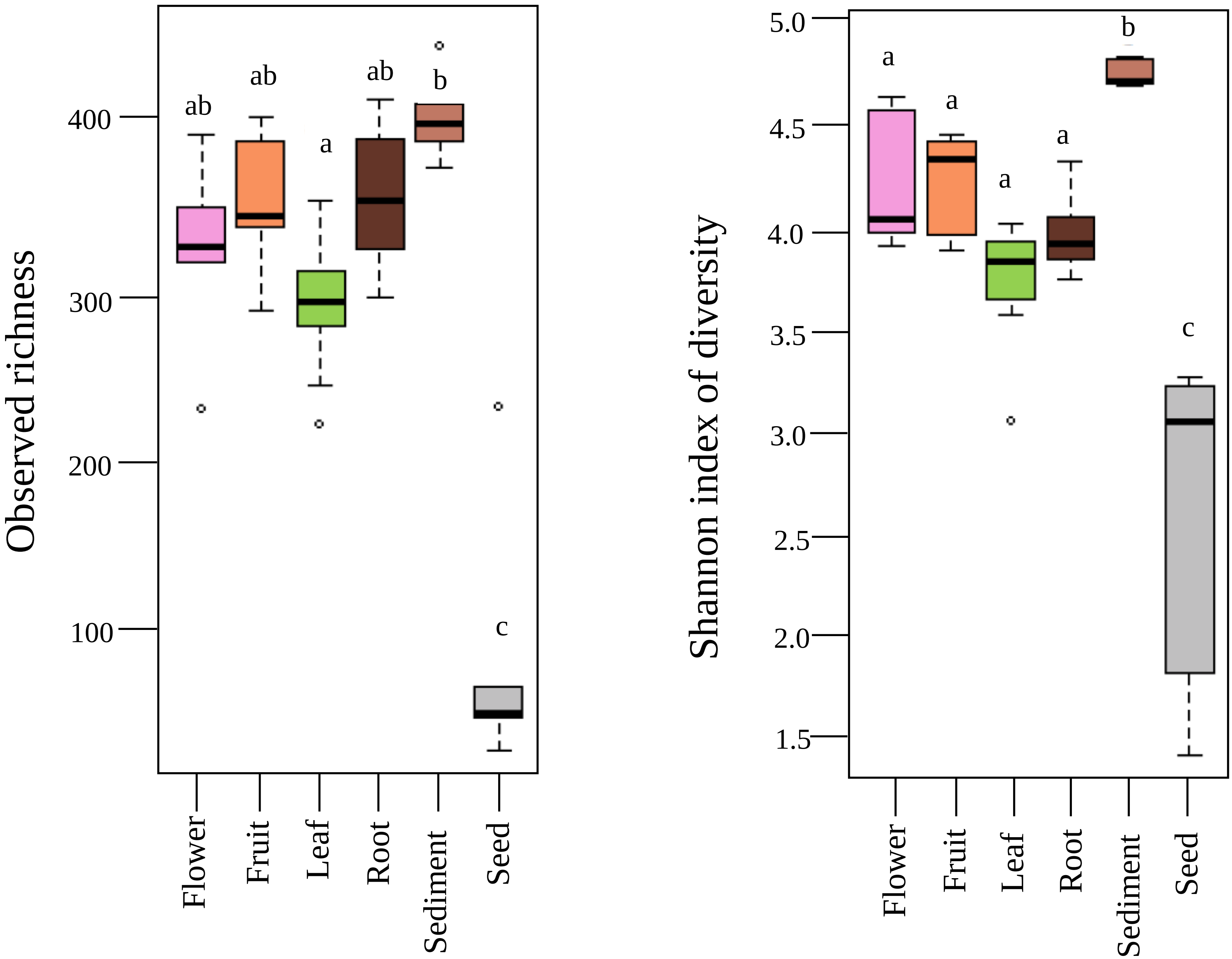

Supplementary Fig.1. Boxplot illustrating alpha diversity indices (number of OTUs observed and Shannon diversity) in bacterial microbiomes of six *H. ovalis* microenvironments. Median values and interquartile ranges have been indicated in the plots. Alpha diversity analyses were based on the total number of sequences per sample which are reported below.

Sample	Num of sequences
Flower_R1	24441
Flower_R2	23709
Flower_R3	31184
Flower_R4	63794
Flower_R5	33007
Fruit_R1	40341
Fruit_R2	17760
Fruit_R3	47249
Fruit_R4	38435
Fruit_R5	33746
Leaf_Time1_R1	44640
Leaf_Time1_R2	17049
Leaf_Time1_R3	32371
Leaf_Time1_R4	31496
Leaf_Time1_R5	22263
Leaf_Time2_R1	28596
Leaf_Time2_R2	34503
Leaf_Time2_R3	26339
Leaf_Time2_R4	29489
Leaf_Time2_R5	60187
Root_Time1_R1	34826
Root_Time1_R2	24624
Root_Time1_R3	47807
Root_Time1_R4	40867
Root_Time1_R5	45109
Root_Time2_R1	25284
Root_Time2_R2	57431
Root_Time2_R3	33808
Root_Time2_R4	68660
Root_Time2_R5	58139
Seed_R1	3479
Seed_R2	4398
Seed_R3	5636
Seed_R4	12205
Seed_R5	713
Sediment_Time1_R1	32317
Sediment_Time1_R2	40870
Sediment_Time1_R3	37566
Sediment_Time1_R4	57599
Sediment_Time1_R5	37062
Sediment_Time2_R1	40813
Sediment_Time2_R2	40665
Sediment_Time2_R3	43883
Sediment_Time2_R4	49085
Sediment_Time2_R5	51558
